# Disease spectrum and comorbidity patterns of malignant neoplasms: a multi-center hospital-based retrospective analysis of inpatient insurance claims data

**DOI:** 10.3389/fonc.2026.1744397

**Published:** 2026-03-27

**Authors:** Meilian Liu, Ling Chen, Peiwen Du, Conghan Wei, Tao Ding, Lizhong Liang, Jiayuan Wu, Zhirong Zeng

**Affiliations:** 1 Department of Pulmonary Oncology, Affiliated Hospital of Guangdong Medical University, Zhanjiang, Guangdong, China; 2 Department of Epidemiology and Health Statistics, School of Public Health, Guangdong Medical University, Zhanjiang, Guangdong, China; 3 School of Ocean and Tropical Medicine, Guangdong Medical University, Zhanjiang, Guangdong, China; 4 Clinical Research Service Center, Affiliated Hospital of Guangdong Medical University, Zhanjiang, Guangdong, China; 5 Institute of Public Health and Wellness, Guangdong Medical University, Zhanjiang, Guangdong, China

**Keywords:** association rule mining, comorbidity profiles, disease spectrum, health insurance data, malignant neoplasms

## Abstract

**Background:**

Previous studies indicate a high comorbidity burden among patients with malignant neoplasms, but claims-based comorbidity patterns have not been systematically characterized at the regional multi-hospital level in China.

**Methods:**

This study is a multi-center, hospital-based retrospective analysis of inpatient insurance claims. We analyzed anonymized inpatient medical insurance claims data (2016–2021) from 163 hospitals in Zhanjiang, China, focusing on hospitalized patients with a primary diagnosis of malignant neoplasms. Malignant neoplasms and comorbidities were identified using International Classification of Diseases, 10th Revision(ICD-10) codes. Disease spectrum was stratified by sex, age, and region. Comorbidity patterns were delineated using association rule mining and network analysis.

**Results:**

Among 107,029 patients, the ten most common malignancies accounted for 75.96% of all cases. Hospitalizations were more frequent among rural populations, males, and individuals aged ≥65 years. The median number of co-diagnosed conditions across major malignancies was 5 (interquartile range [IQR]: 3–7). Network analysis revealed three major co-diagnosis clusters: 1) liver cancer with chronic viral hepatitis and hepatic fibrosis; 2) lung cancer with chronic obstructive pulmonary disease (COPD) and pneumonia; and 3) colorectal cancer with inflammatory bowel disease-related conditions and intestinal obstruction. Patterns varied across sex, age groups, and urban–rural residence.

**Conclusions:**

This study demonstrates a high comorbidity burden among hospitalized cancer patients, with distinct malignancy-specific co-diagnosis patterns. These findings support the need for integrated clinical management and targeted healthcare resource allocation, particularly for older, male, and rural patient populations.

## Introduction

Malignant neoplasms are a major global public health issue and a leading cause of death. According to the 2021 global cancer data from the International Cancer Center, there were 19.29 million new cancer cases and nearly 10 million cancer deaths worldwide in 2020 (1). China accounted for 4.57 million new cases, representing 23.7% of the global total cases, and the global cancer burden is projected to reach 28.4 million cases by 2040. In China, cancer incidence and mortality continue to increase, with 4,824,700 new cases and 2,574,200 deaths reported in 2022 (2).

Malignant neoplasms arise from interactions of genetic, environmental, behavioral, and socioeconomic risk factors. Age significantly influences cancer development, with most (>50%) cases occurring in adults >65 years (3). Urban-rural disparities in cancer incidence reflect differential environmental exposures, lifestyle practices, and healthcare access (4), contributing to superior early diagnosis/treatment rates in urban settings due to concentrated resources (5). South China, especially Guangdong Province, exhibits distinct epidemiologic pattern of cancer driven by HBV/HCV prevalence, EBV endemicity, and dietary exposures (6). Zhanjiang City exhibits a strikingly elevated age-standardized incidence rate (ASIR) of liver cancer 2.1 times the national average (7), warranting targeted investigation.

Comorbidity, defined as the presence of two or more concurrent chronic conditions, is highly prevalent, especially in middle-aged and older adults (8). In cancer patients, comorbidities significantly impact quality of life (9–11), functional status (9, 12), mortality risk (13, 14), and healthcare costs (15, 16), placing considerable strain on healthcare systems. Optimizing comorbidity management in cancer patients is essential for clinical decision-making and prognosis improvement. The rising global cancer burden, coupled with population aging, underscores the increasing importance of understanding and addressing comorbidities in oncology care.

Cancer-related multimorbidity is increasingly recognized as a major determinant of treatment tolerance, prognosis, and healthcare utilization. However, evidence from China remains limited and is often constrained to single-center cohorts or predefined comorbidity indices. Using region-wide inpatient insurance claims from 163 hospitals in Zhanjiang (2016–2021), this study provides a comprehensive characterization of the inpatient malignant neoplasm spectrum and claims-based co-diagnosis patterns. By applying association rule mining and network analysis, and further exploring heterogeneity by sex, age, and urban-rural residence, our findings may inform integrated inpatient management strategies and local resource allocation for clinically complex cancer populations.

Research on cancer comorbidities in China remains nascent, limiting evidence-based clinical management strategies for malignant neoplasms. Medical insurance claims data provide an effective method for identifying comorbidities within cancer populations (17, 18), overcoming inaccuracies inherent in self-reported data (19). As comorbidities in administrative databases derive from clinical diagnoses, this approach enhances the quality of epidemiological research.

This study seeks to:

1. Characterize the cancer spectrum in Zhanjiang utilizing population-level insurance claims data (2016-2021);
2. Identify comorbidity patterns through association rule mining;
3. Investigate variations in comorbidity patterns according to sex, age, and urban-rural residence.

## Materials and methods

### Data source

The study utilized anonymized medical insurance claims data covering all inpatient admissions with a primary diagnosis of malignant neoplasm between January 1, 2016, and December 31, 2021. The data originated from 163 hospitals located in Zhanjiang, China (**Supplementary Table 1**). Available variables included age, sex, urban–rural residence, year of admission, and diagnoses coded according to the International Classification of Diseases, 10th Revision (ICD-10). The operational definitions of all comorbid conditions, including the corresponding ICD-10 codes, are detailed in **Supplementary Table 2** to ensure transparency and reproducibility. Data processing was governed by the following principles: 1. ICD-10 standardization; 2. Deduplication by patient ID; 3. Temporal linkage of multi-admission records.

### Study subjects

**Figure 1:**
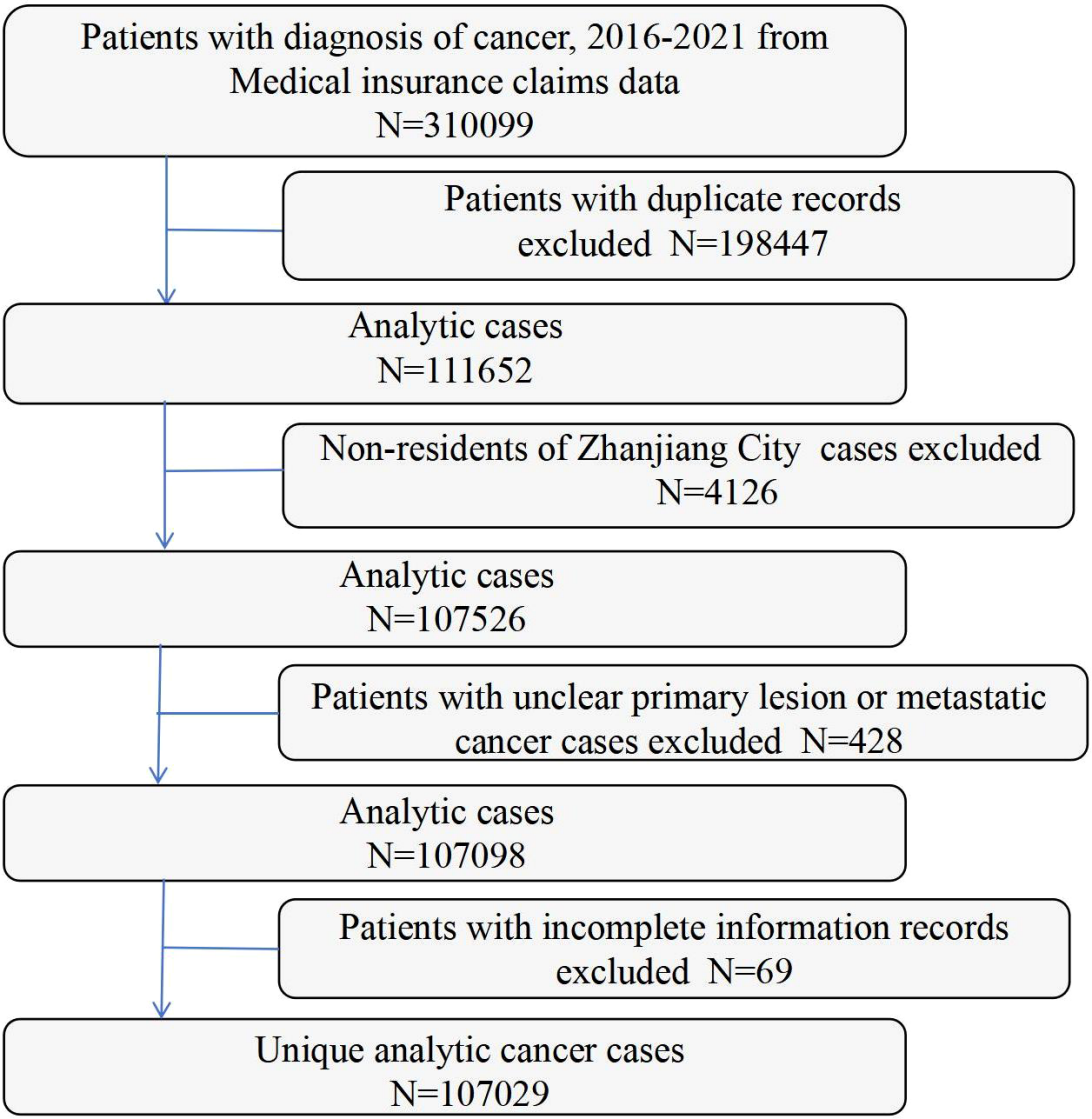
Patient identification flowchart. (n = 107,029).

Inclusion criteria comprised: 1. Hospitalized patients with a primary diagnosis of malignant neoplasm during the study period; 2. Complete information on primary diagnosis, age, sex, residence, and admission date; 3. Malignant neoplasms with a defined primary site. Exclusion criteria were: 1. Secondary malignant neoplasms; 2. Malignant neoplasms of unspecified or unknown primary site; 3. Non-local residents. A final cohort of 107,029 patients met these criteria (**Figure 1**).

### Comorbidity ascertainment

In this claims-based study, comorbidity was operationally defined as any additional diagnosis co-recorded with the index malignant neoplasm in inpatient claims. Accordingly, the term refers to claims-based co-diagnosed conditions during hospitalization, encompassing pre-existing chronic diseases, cancer-related manifestations, treatment-related adverse events, and in-hospital complications. Diagnoses across multiple admissions were aggregated, while conditions recorded only once and not re-documented within 6 months were excluded to reduce transient diagnoses. Accordingly, the identified patterns reflect clinically meaningful co-diagnosis clusters rather than causal relationships.

### Statistical analysis

Descriptive statistics characterized the patient cohort and disease spectrum, stratified by sex, age group (0–19, 20–39, 40–64, ≥65 years), and urban-rural residence. Comorbidity burden was described using median counts and interquartile ranges (IQR), and prevalence percentages for the top malignancies. The average annual growth rate (AAGR) was calculated as:


$$\text{AAGR}={\left(\frac{V_{t}}{V_{0}}\right)}^{\frac{1}{t}}-1$$

where *V_0_* and *V_t_* denote the annual number of hospitalizations in the first (2016) and last (2021) year (20), respectively, and *t* denotes the number of years between them. This formulation is consistent with standard compound annual growth rate calculations used in epidemiological trend descriptions (21).Network construction parameters, including support, confidence, and lift thresholds, were fixed across analyses to ensure reproducibility.

### Association rule mining

We applied the Apriori algorithm to identify frequent co-diagnosis itemsets and generate association rules (22). This algorithm employs an iterative, level-wise search strategy to construct candidate itemsets and prunes those that fail to meet a predefined minimum support threshold, based on the principle that all subsets of a frequent itemset must themselves be frequent. This property reduces computational burden while ensuring robust pattern identification.

Association rules were evaluated using three standard metrics: support, confidence, and lift. Support (range: 0–1) represents the proportion of records containing a given itemset and reflects its prevalence within the dataset. Confidence (range: 0–1) denotes the conditional probability of observing the consequent diagnosis given the antecedent diagnosis, thereby quantifying the strength of the directional association. Lift (range: >0) compares the observed co-occurrence frequency with that expected under statistical independence; lift > 1 indicates a positive association, lift = 1 indicates independence, and lift< 1 suggests a negative association.

Hierarchical clustering with complete linkage was performed based on pairwise Jaccard similarity matrices to identify clusters of comorbid conditions (23). Significant association rules were visualized using directed network graphs, in which nodes represented malignant neoplasms and co-diagnosed conditions, directed edges denoted associations, edge thickness was proportional to rule confidence, and edge color intensity corresponded to lift magnitude.

### Software

The Apriori algorithm was implemented using the “arules” package in R (version 4.2.0). Other statistical analyses, including descriptive statistics, were performed using SPSS (version 26.0). Statistical significance was set at *P<* 0.05.

## Results

### Patient characteristics

The study cohort comprised 107,029 patients with primary malignant neoplasms. Males constituted 55.89% (n = 59,814) and females 44.11% (n = 47,215), yielding a male-to-female ratio of 1.27:1. Urban residents accounted for 71.98% (n = 79,551) and rural residents for 28.02% (n = 30,960). Age ranged from 0 to 109 years (mean age, 62.47 years), with the largest proportion (47.94%) aged ≥65 years, followed by 40–64 years (43.65%), 20–39 years (7.06%), and 0–19 years (1.34%). (data not shown).

### Disease spectrum composition

**Figure 2:**
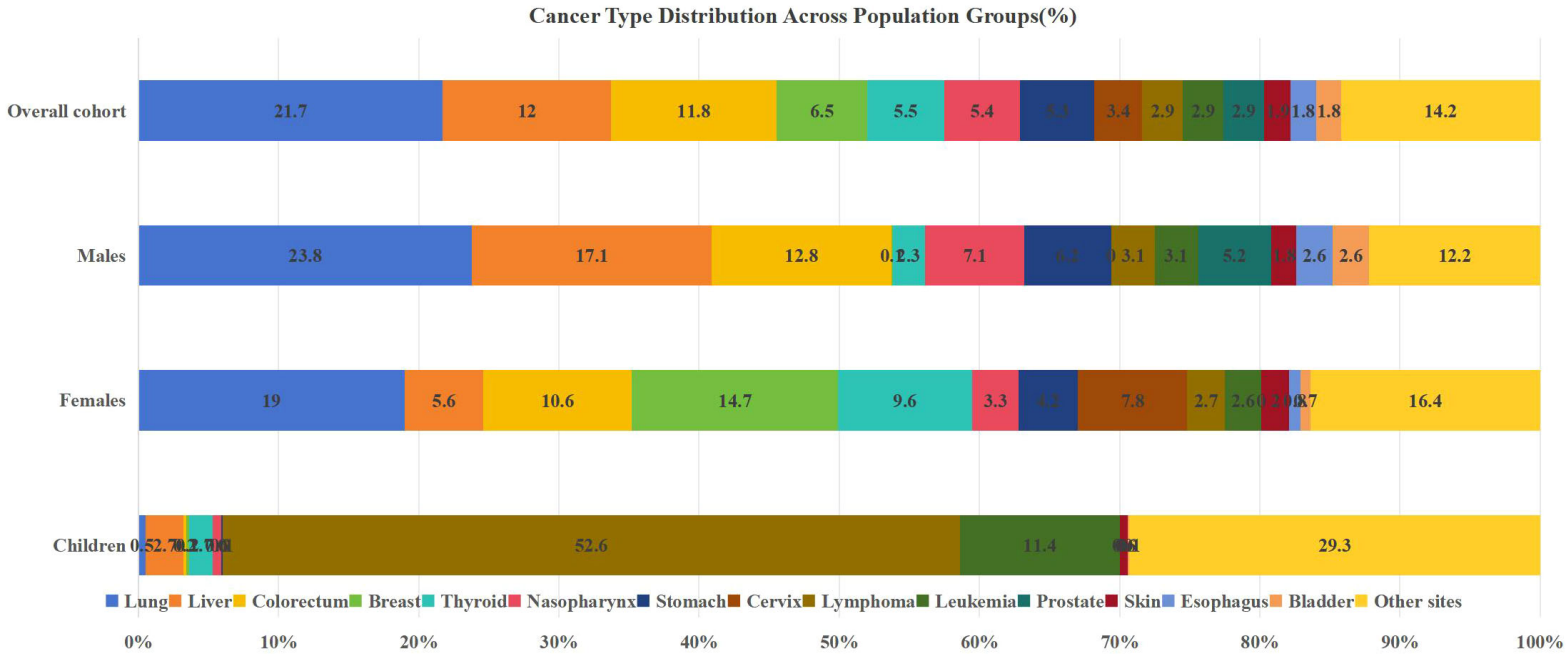
Proportion of Malignant tumors composition in different population groups.

Within the cohort of 107,029 patients, a total of 110,511 primary malignant tumors were diagnosed, reflecting cases with multiple primary malignancies. Lung cancer was the most frequent diagnosis (n=23,202, 21.00%). Subsequent malignancies comprised liver, colorectal, breast, thyroid, nasopharyngeal (NPC), gastric, cervical cancers, lymphoma, and leukemia-collectively constituting 75.96% of cases. Gender stratification showed lung, liver, colorectal, NPC, and gastric cancers were predominant in males (collectively 64.63% of male cases), while lung, breast, colorectal, thyroid, and cervical cancers were most frequent in females (collectively 59.95% of female cases). Among pediatric patients (<20 years), leukemia, brain/CNS neoplasms, and lymphoma were predominant (71.93%)(**Table 1**; **Figure 2**).

**Table 1:** The composition of common malignant tumors in Zhanjiang City from 2016 to 2021.

ICD10	Site	All			Men			Women		
		Cases	Constituent ratio(%)	Cumulative proportion	Cases	Constituent ratio(%)	Cumulative proportion	Cases	Constituent ratio(%)	Cumulative proportion
C33-34	Lung	23202	21.00	21.00	14229	12.88	12.88	8973	8.12	8.12
C22	Liver	12886	11.66	32.66	10227	9.25	22.13	2659	2.41	10.53
C18-20	Colorectum	12657	11.45	44.11	7640	6.91	29.04	5017	4.54	15.07
C50	Breast	6956	6.29	50.40	38	0.03	29.08	6918	6.26	21.33
C73	Thyroid	5897	5.34	55.74	1346	1.22	30.30	4551	4.12	25.44
C11	Nasopharynx	5785	5.23	60.97	4220	3.82	34.11	1565	1.42	26.86
C16	Stomach	5673	5.13	66.11	3700	3.35	37.46	1973	1.79	28.65
C53	Cervix	3677	3.33	69.43	–	–	37.46	3677	3.33	31.97
C81-85	Lymphoma	3616	3.27	72.71	2183	1.98	39.44	1433	1.30	33.27
C91-95	Leukemia	3596	3.25	75.96	2149	1.94	41.38	1447	1.31	34.58
C61	Prostate	3121	2.82	78.78	3121	2.82	44.21	–	–	34.58
C44	Skin	2046	1.85	80.64	1097	0.99	45.20	949	0.86	35.44
C15	Esophagus	1933	1.75	82.39	1536	1.39	46.59	397	0.40	35.80
C67	Bladder	1898	1.72	84.10	1546	1.40	47.99	352	0.32	36.11
	Other sites	17568	15.90	100	8880	8.04	56.02	8688	7.86	43.98
ALL	All sites	110511	100	100	61912	56.02	56.02	48599	43.98	43.98

### Temporal trends

Annual hospitalizations for malignant neoplasms increased from 16,470 in 2016 to 18,466 in 2021, representing an average annual growth rate of 2.32%. Lung cancer consistently had the highest incidence among newly diagnosed cases each year. Liver, colorectal, breast, NPC, thyroid, gastric, and cervical cancers consistently ranked among the top 10 malignancies throughout the six years. Thyroid cancer showed the most notable rise in rank, moving from 7th position in 2016 to 4th by 2021 (**Table 2**).

**Table 2:** Changes in the incidence composition and ranking of major malignant tumors in Zhanjiang City from 2016 to 2021.

Site	2016	2017	2018	2019	2020	2021	Change in rank
Lung	3655 (1)	3288 (1)	3569 (1)	3794 (1)	3851 (1)	4064 (1)	0
Liver	2168 (2)	1995 (2)	2119 (2)	2032 (3)	2027 (3)	1908 (3)	-1
Colorectum	1752 (3)	1822 (3)	2039 (3)	2178 (2)	2108 (2)	2242 (2)	1
Breast	982 (4)	887 (5)	1307 (4)	1145 (4)	1163 (4)	1319 (5)	-1
Thyroid	674 (7)	644 (7)	878 (7)	936 (6)	1020 (5)	1543 (4)	3
Nasopharynx	924 (6)	878 (6)	1115 (5)	992 (5)	842 (6)	837 (6)	0
Stomach	947 (5)	905 (4)	892 (6)	901 (7)	840 (7)	872 (7)	-2
Cervix	562 (8)	540 (8)	642 (8)	605 (8)	590 (8)	604 (8)	0
Lymphoma	474 (10)	401 (10)	453 (10)	473 (11)	463 (11)	524 (10)	0
Leukemia	494 (9)	502 (9)	508 (9)	494 (10)	490 (10)	506 (11)	-2

### Demographic variations

**Figure 3:**
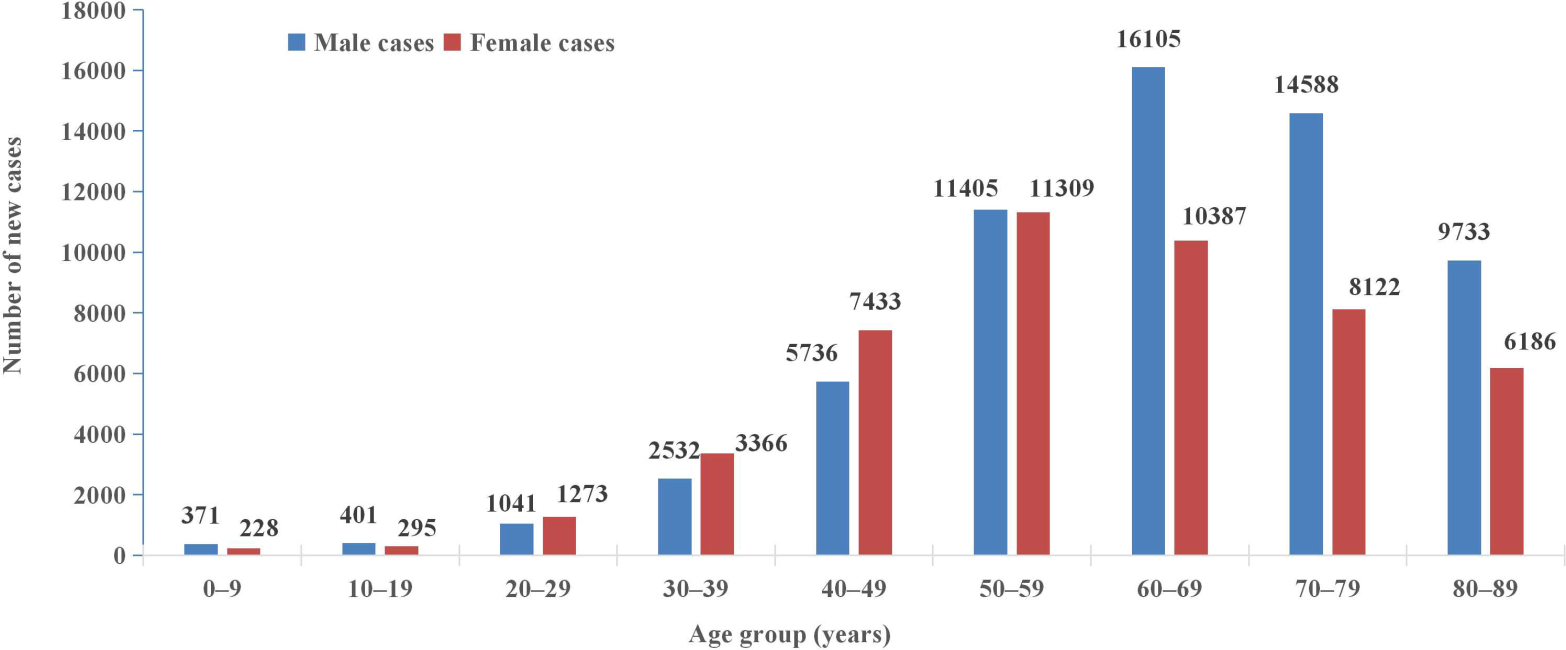
Age-specific incidence of new cancer cases by sex. The number of cases among women under 50 was higher than that of men, while the opposite was true for those over 50. The peak age group was 50–59 years for females and 60–69 years for males.

**Figure 4:**
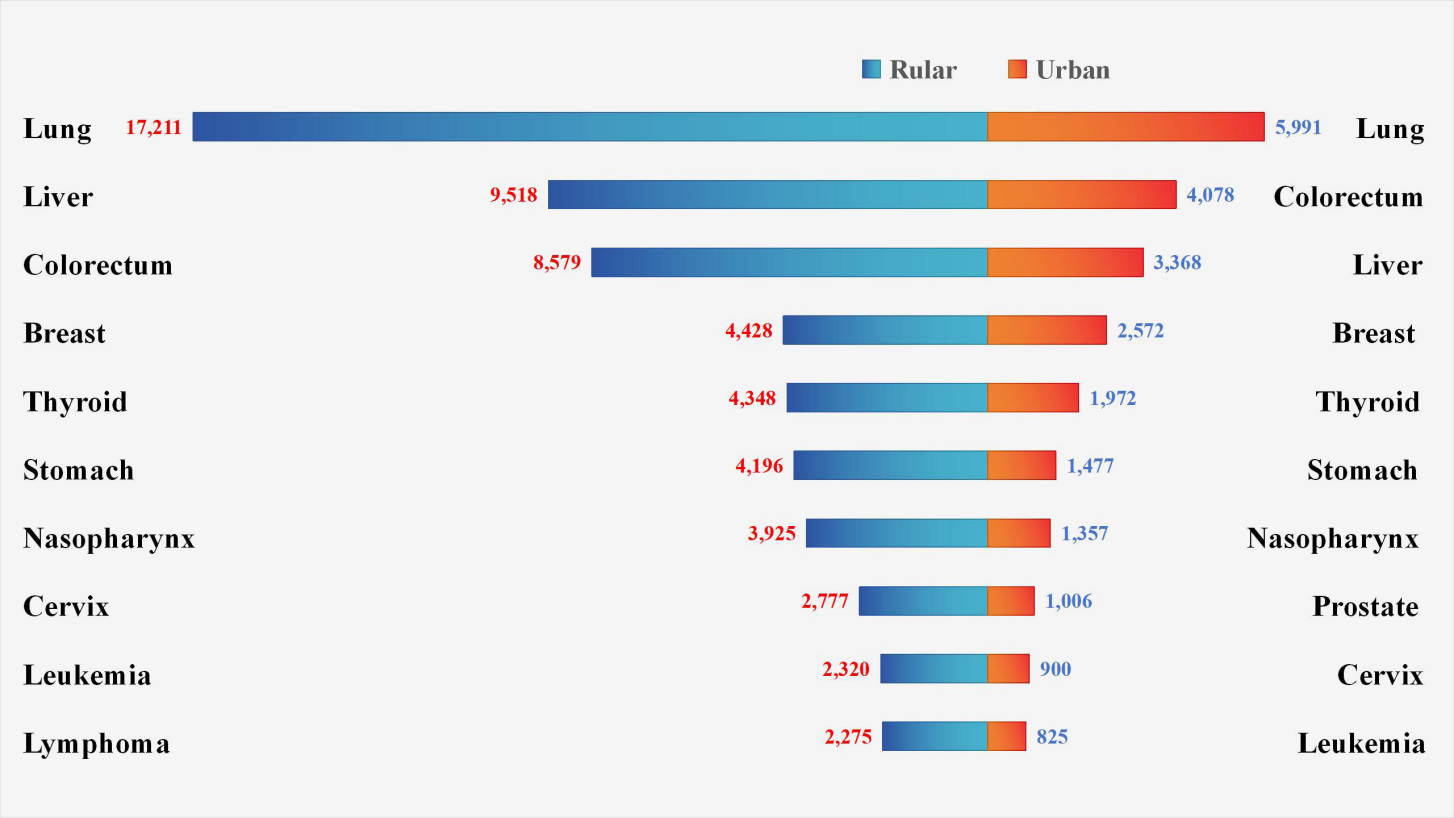
Distribution of new cancer cases by urban-rural residence. Lung cancer was the most prevalent malignant tumor in both urban and rural areas. Liver cancer ranked second, with lymphoma also identified as a common malignancy in rural regions. Colorectal cancer was the second common cancer, alongside prostate cancer as another frequently observed malignancy in urban populations.

Over the study period, 61,912 new malignancies were diagnosed in males compared to 48,599 in females. Females showed higher hospitalization counts than males below 50 years, whereas males predominated from 50 years onward. The peak age group was 50–59 years in females and 60–69 years in males (**Figure 3**). Lung cancer was the most common malignancy in both urban and rural areas. Among rural residents, liver cancer ranked second, followed by colorectal cancer. In urban areas, colorectal cancer was the second most common, followed by liver cancer (**Figure 4**).

### Comorbidity burden

The median number of comorbidities varied significantly across cancer types. Patients with lung cancer (median 6, IQR 4–7), liver cancer (6, IQR 3–8), and NPC (6, IQR 3–10) bore the heaviest comorbidity burden. Colorectal, gastric, and lymphoma cancers had a median of 5 comorbidities (IQR 3–7). Cervical cancer patients had a median of 4 comorbidities (IQR 2–7). Breast cancer (3, IQR 1–6), leukemia (3, IQR 2–5), and thyroid cancer (3, IQR 2–5) had the lowest median comorbidity counts. The prevalence of at least one comorbidity among these top malignancies ranged from 69.6% in thyroid cancer to 97.3% in liver cancer. (data not shown).

### Comorbidity patterns

**Figure 5:**
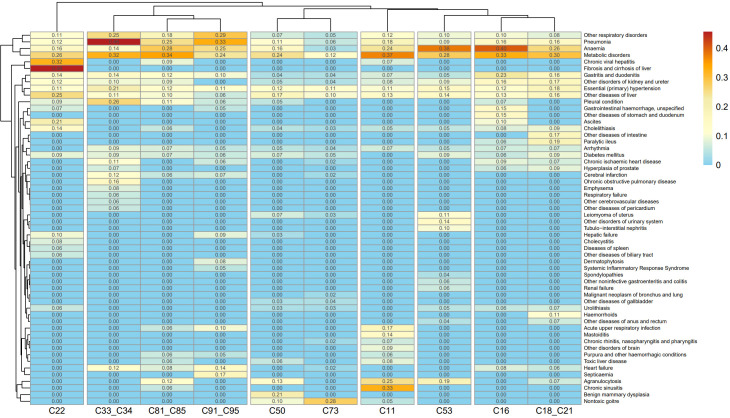
Heatmap of top 20 comorbidities for the ten most common malignancies (cluster analysis). This cluster analysis chart utilized a heat map format to display the clustering of comorbid conditions among the top 20 rankings of 10 common malignant neoplasms. The main features we used included the types of cancer, the types of comorbidities, and the prevalence rates of both. Detailed ICD-10 code definitions for comorbidity categories are provided in **Supplementary Table 2**.

Binary comorbidity analysis identified 93 patterns exceeding 10% prevalence across ten common malignancies. Liver cancer demonstrated the highest comorbidity rate: 46.7% with concurrent liver fibrosis/cirrhosis, followed by pneumonia in lung cancer (45.6%). Other frequently co-occurring comorbidities included pleural disease, hypertension, COPD, anemia, diabetes, ischemic heart disease, and cerebral infarction. The top 20 most frequent comorbidities for each of the 10 most prevalent malignancies are presented in **Figure 5** and **Supplementary Table 3**.

**Figure 6:**
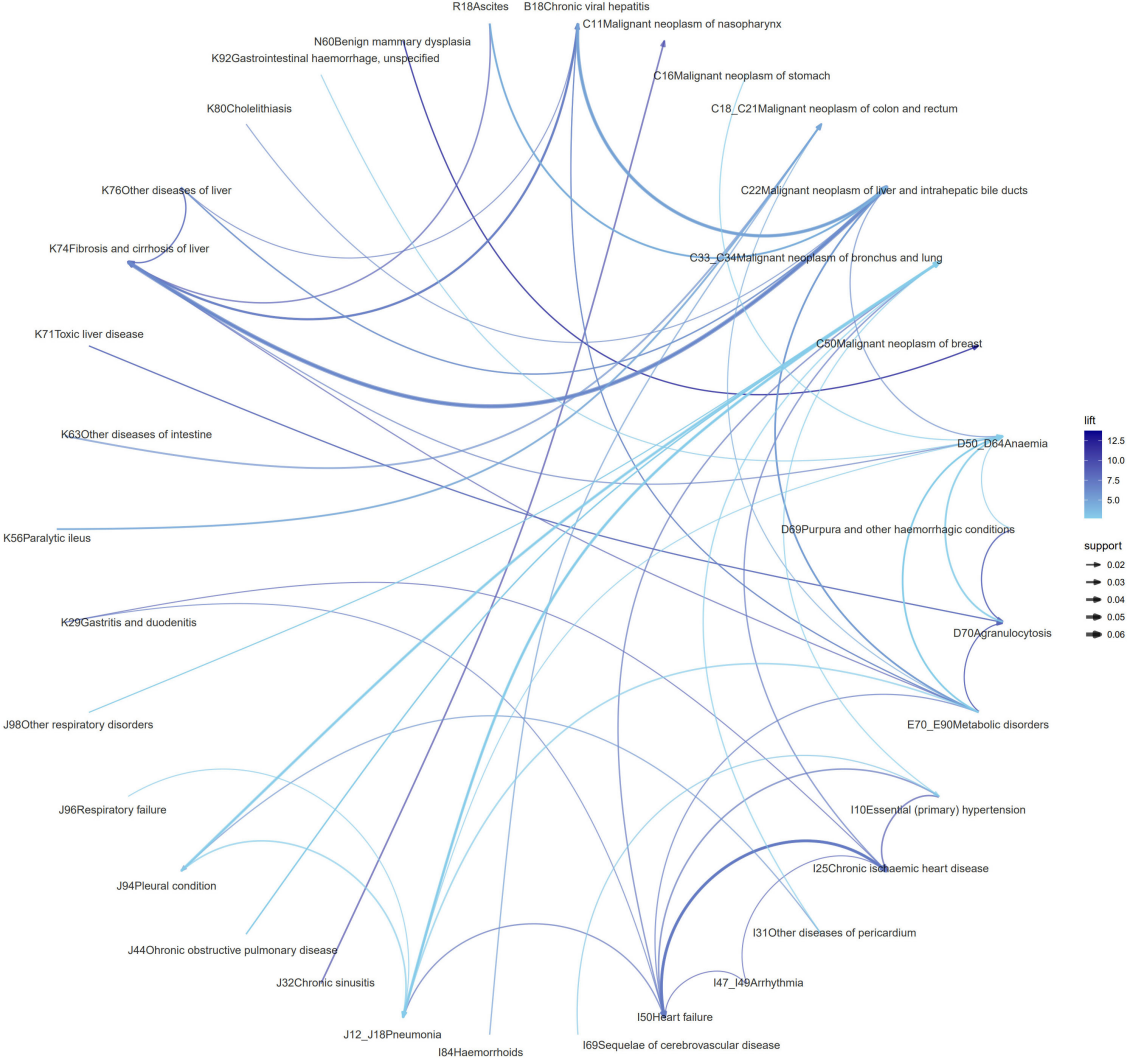
Network analysis of comorbidity patterns. The comorbidity network diagram was created using association rule mining techniques to illustrate the relationships between various diseases. Nodes represent malignant neoplasms and co-diagnosed conditions. Directed edges indicate association rules derived from the Apriori algorithm. Edge thickness is proportional to rule confidence, and edge color intensity reflects lift magnitude. Only association rules meeting predefined thresholds (support, confidence, and lift) are displayed.

Association rule mining yielded 138 significant rules, with particularly high burdens observed in lung, liver, and colorectal cancer patients. Key associations included a robust link between COPD and pleural lesions in lung cancer (lift = 3.30), a striking association of chronic viral hepatitis with liver fibrosis/cirrhosis in liver cancer (lift = 6.95), and a pronounced association between chronic intestinal inflammatory disease and colorectal cancer (lift = 5.94). Beyond these major malignancies, NPC showed strong links to both chronic intestinal inflammatory disease and chronic sinusitis (lift = 9.76), while benign breast hyperplasia demonstrated a remarkably robust association with breast cancer (lift = 13.72). The full spectrum of comorbidity patterns across all cancers is visualized in the network graph (**Figure 6**). Cancer-specific network details are provided in Supplementary 3–12 Figure.

**Figure 7:**
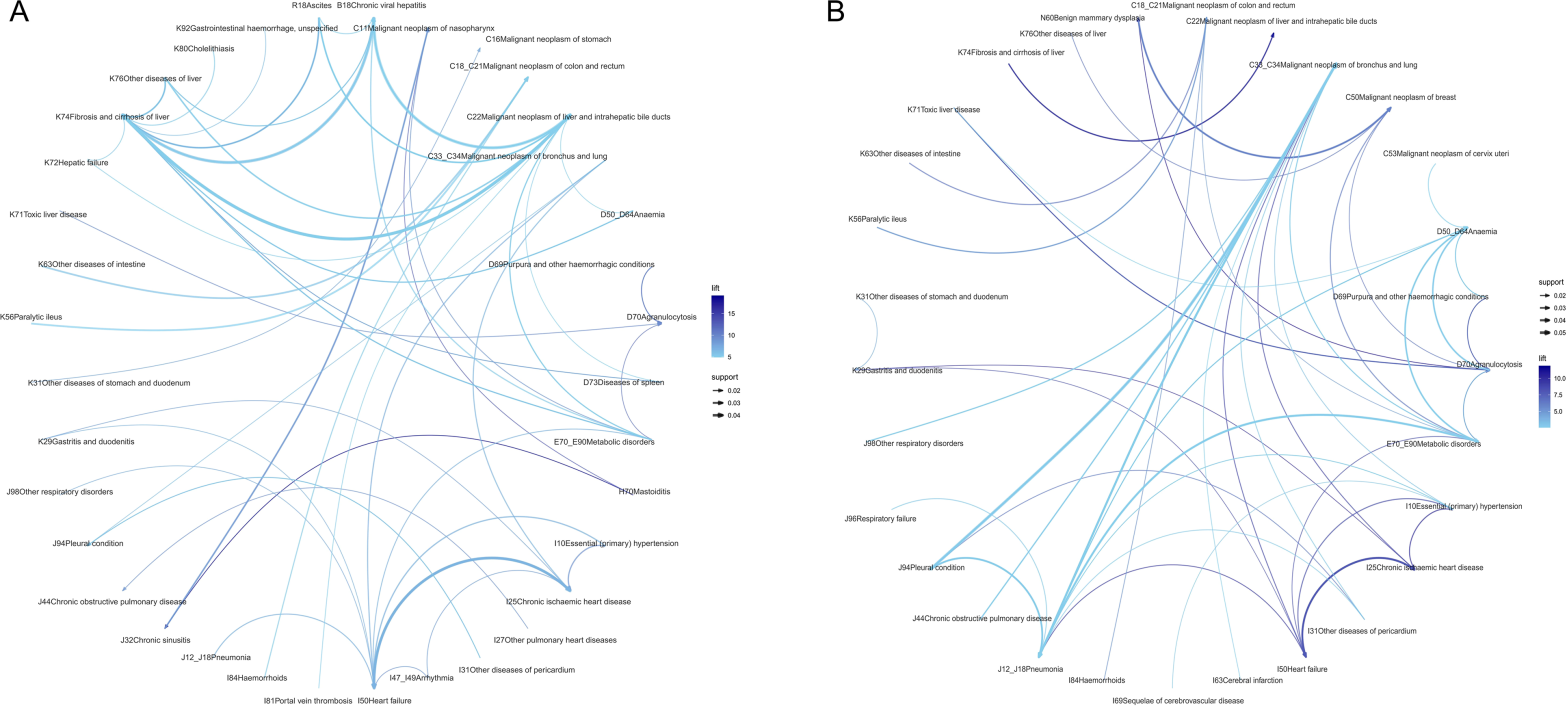
Sex-specific comorbidity patterns of patients with malignant neoplasms. **(A)** Male patients. **(B)** Female patients. Network construction procedures, association rule mining parameters, and visualization conventions (nodes representing diagnoses, edge thickness proportional to confidence, and edge color intensity corresponding to lift) were identical to those described in **Figure 6**.

Sex-specific comorbidity patterns are summarized in **Table 3**. Anemia and metabolic disorders constituted shared comorbidities across both sexes. Males frequently manifested hepatic fibrosis, chronic hepatitis with liver cancer, and pleural disease with lung cancer, alongside pneumonia (**Figure 7A**). Females predominantly presented with pleural disease in lung cancer, chronic ischemic heart disease with heart failure, and benign breast hyperplasia with breast cancer (**Figure 7B**).

**Table 3:** The distribution of comorbidities associated with cancer patients in males and females.

Sex	Rules	Support (%)	Confidence (%)	lift
Male				
	Anemia=> Metabolic disorders	11.71	56.87	1.78
	Fibrosis and cirrhosis of liver => Liver cancer	9.81	90.82	4.81
	Chronic viral hepatitis => Liver cancer	7.88	72.78	3.86
	Pleural condition => Lung cancer	7.04	61.47	2.38
	Pleural condition => Pneumonia	6.52	56.90	2.20
	Lung cancer, Metabolic disorders => Pneumonia	5.04	58.44	2.26
	Chronic obstructive pulmonary disease => Lung cancer	4.96	56.52	2.19
	CVH, Fibrosis and cirrhosis of liver=> Liver cancer	4.93	95.63	5.07
	Pneumonia, Pleural condition=> Lung cancer	4.46	68.38	2.64
	Anemia, Pneumonia => Metabolic disorders	4.41	67.69	2.12
Female				
	Anemia=> Metabolic disorders	12.66	52.58	1.65
	Pleural condition => Lung cancer	5.91	59.49	2.70
	Pleural condition => Pneumonia	5.47	55.05	2.51
	Agranulocytosis => Metabolic disorders	5.32	57.99	1.82
	Lung cancer, Metabolic disorders => Pneumonia	4.28	56.73	2.59
	Anemia, Pneumonia => Metabolic disorders	4.12	67.68	2.12
	Chronic ischemic heart disease => Heart failure	3.74	56.55	7.84
	Benign mammary dysplasia => Malignant neoplasm of breast	3.73	77.39	5.89
	Pneumonia, Pleural condition=>Lung cancer	3.62	66.21	3.00
	Anemia, Agranulocytosis =>Metabolic disorders	3.37	68.67	2.15

CVH, chronic viral hepatitis.

**Figure 8:**
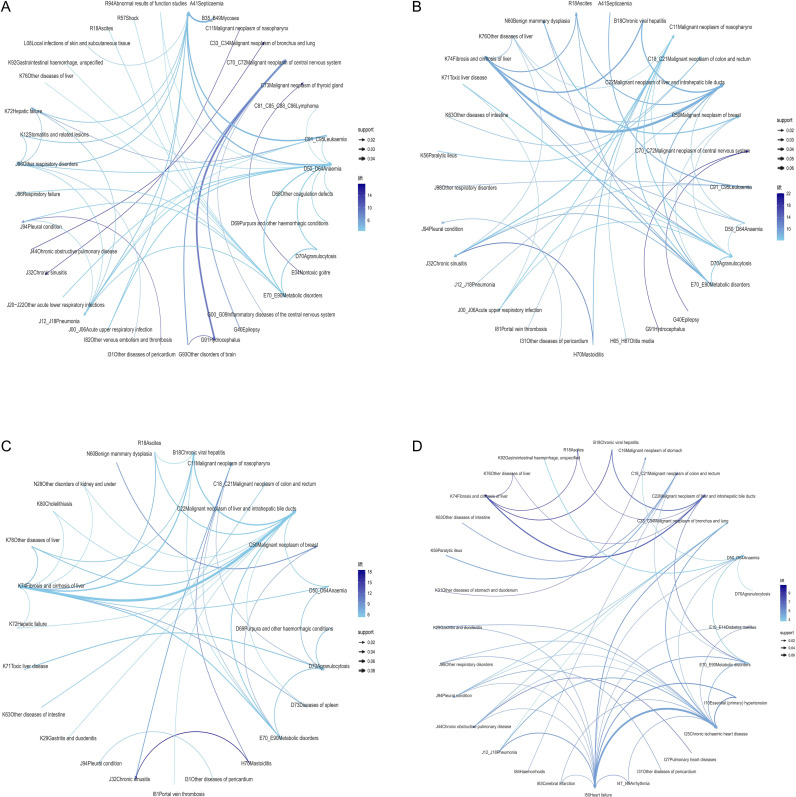
Age-specific comorbidity patterns among patients with malignant neoplasms. **(A)**<20 years. **(B)** 20–39 years. **(C)** 40–64 years. **(D)** ≥65 years. All networks were constructed using the same analytical framework, association rule mining thresholds, and visualization settings as detailed in **Figure 6**.

Age-specific comorbidity profiles exhibited considerable variation (**Table 4**). Among patients aged<20 years, comorbidities primarily centered on leukemia and included anemia, pneumonia, and other respiratory disorders (**Figure 8A**). The 20–39 age group was characterized by predominant associations of liver cancer with hepatitis and thyroid cancer with goiter (**Figure 8B**). Common patterns in the 40–64 age cohort involved anemia with metabolic disorders, hepatitis, and pleural disease occurring in lung cancer patients (**Figure 8C**). For patients aged ≥65 years and older, prevalent comorbidities comprised heart failure with chronic ischemic heart disease, COPD (particularly in lung cancer), and diabetes with hypertension (**Figure 8D**).

**Table 4:** The distribution of comorbidities associated with cancer in different age groups.

Age	Rules	Support (%)	Confidence (%)	lift
0-19						
	Anemia=> Leukaemia	14.23	50.96	1.21
	Pneumonia=> Leukaemia	12.75	54.60	1.29
	Septicemia=> Leukaemia	11.95	82.41	1.95
	Other respiratory disorders=> Leukaemia	9.26	53.49	1.27
	Acute upper respiratory infection=> Leukaemia	8.99	69.79	1.65
	Septicemia, Pneumonia=> Leukaemia	5.10	92.68	2.19
	Septicemia, Anemia=> Leukaemia	5.10	80.85	1.91
	Hepatic failure=> Leukaemia	5.10	73.08	1.73
	Anemia, Pneumonia=> Leukaemia	4.97	53.62	1.27
	Hydrocephalus=>Malignant neoplasm of CNS	4.56	97.14	9.78
20-39						
	Agranulocytosis => Metabolic disorders	6.25	53.95	1.93
	Chronic viral hepatitis=> Liver cancer	6.16	56.23	4.46
	Fibrosis and cirrhosis of liver => Liver cancer	6.09	93.91	8.57
	Nontoxic goiter => Malignant neoplasm of thyroid gland	5.32	68.36	4.37
	Chronic sinusitis=>NPC	4.96	73.02	5.96
	Fibrosis and cirrhosis of live=> Chronic viral hepatitis	3.77	58.17	4.61
	Ascites => Metabolic disorders	3.72	62.16	2.22
	CVH, Fibrosis and cirrhosis of liver => Liver cancer	3.61	95.71	8.73
	Ascites=> Liver cancer	3.00	50.15	4.58
	Benign mammary dysplasia => Breast cancer	2.73	66.38	6.97
40-64						
	Anemia => Metabolic disorders	12.48	54.74	1.70
	Fibrosis and cirrhosis of liver => Liver cancer	8.93	91.62	5.72
	Chronic viral hepatitis=> Liver cancer	7.23	66.81	4.17
	Pleural condition=> Lung cancer	5.06	54.60	2.87
	CVH, Fibrosis and cirrhosis of liver => Liver cancer	4.64	95.14	5.94
	Anemia, Pneumonia =>Metabolic disorders	4.01	70.24	2.19
	Ascites=> Liver cancer	3.45	55.23	3.45
	Pneumonia, Pleural condition=> Lung cancer	3.22	63.19	3.32
	Chronic sinusitis=>NPC	3.14	62.55	7.40
	Benign mammary dysplasia =>Breast cancer	2.81	79.73	8.88
≥65						
	Heart failure => Chronic ischemic heart disease	7.21	59.60	4.71
	Chronic obstructive pulmonary disease => Lung cancer	6.96	57.52	1.81
	Pneumonia, Pleural condition=> Lung cancer	5.38	72.80	2.29
	Diabetes mellitus =>Essential (primary) hypertension	5.21	50.44	1.89
	Fibrosis and cirrhosis of liver => Liver cancer	4.68	83.97	6.92
	Essential (primary) hypertension, Pneumonia=> Lung cancer	4.04	52.50	1.65
	Essential hypertension, Heart failure=>Chronic heart disease	3.43	70.39	5.56
	Paralytic ileus=> Colorectal cancer	3.32	66.81	4.58
	Chronic viral hepatitis=> Liver cancer	3.22	73.36	6.05
	Other diseases of intestine=> Colorectal cancer	2.67	78.37	5.37

CNS, central nervous system; NPC, nasopharyngeal carcinoma; CVH, chronic viral hepatitis.

**Figure 9:**
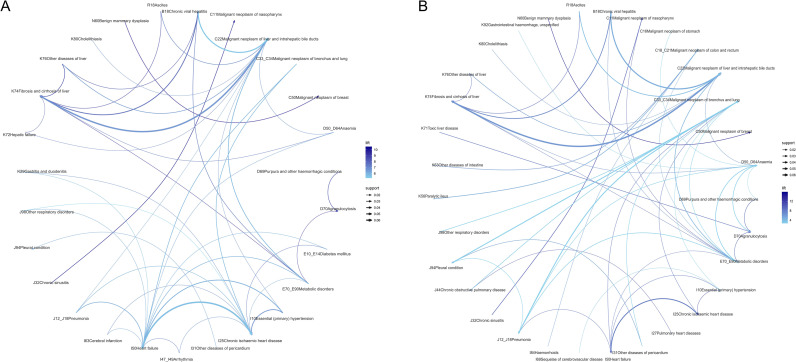
Urban–rural comorbidity patterns among patients with malignant neoplasms. **(A)** Urban residents. **(B)** Rural residents. Network construction methods, association rule parameters, and visualization conventions were consistent with those described in **Figure 6**.

Stratified analysis revealed distinct urban-rural disparities in comorbidity patterns (**Table 5**). Urban residents exhibited higher frequencies of pleural disease in lung cancer, chronic ischemic heart disease with heart failure, and benign breast hyperplasia in breast cancer (**Figure 9A**). Conversely, rural populations showed heightened associations of liver fibrosis with liver cancer, chronic hepatitis with liver cancer, and pneumonia with lung cancer (**Figure 9B**).

**Table 5:** Results of association rules for diseases of different region.

Region	Rules	Support (%)	Confidence (%)	lift
Urban				
	Anemia=> Metabolic disorders	12.18	55.73	1.73
	Fibrosis and cirrhosis of liver => Liver cancer	6.38	88.27	6.83
	Pleural condition => Lung cancer	5.85	57.63	2.53
	Heart failure=> Chronic ischemic heart disease	5.71	57.75	5.76
	Pleural condition => Pneumonia	5.67	55.92	2.42
	Chronic ischemic heart disease => Essential hypertension	5.37	53.52	2.20
	Chronic viral hepatitis=> Liver cancer	4.91	68.01	5.26
	Anemia, Pneumonia => Metabolic disorders	4.40	69.14	2.15
	Cerebral infarction =>Essential hypertension	4.13	52.17	2.14
	Pneumonia, Pleural condition=>Lung cancer	3.67	64.65	2.8
Rural				
	Anemia=> Metabolic disorders	12.09	54.56	1.71
	Pleural condition => Lung cancer	6.83	61.78	2.49
	Fibrosis and cirrhosis of liver => Liver cancer	6.63	89.06	6.39
	Pleural condition=>Pneumonia	6.22	56.27	2.29
	Chronic viral hepatitis=> Liver cancer	5.21	66.26	4.76
	Pneumonia, Pleural condition=>Lung cancer	4.27	68.56	2.76
	Anemia, Pneumonia => Metabolic disorders	4.24	67.13	2.11
	Chronic obstructive pulmonary disease => Lung cancer	4.20	57.27	2.31
	Chronic ischemic heart disease => Heart failure	3.50	54.85	7.87
	CVH, Fibrosis and cirrhosis of liver => Liver cancer	3.22	95.27	6.84

CVH, chronic viral hepatitis.

## Discussion

### Key findings

This large, population-based analysis utilizing comprehensive medical insurance data provides a detailed characterization of the malignant neoplasm spectrum and associated comorbidity patterns among hospitalized patients. We identified lung, liver, and colorectal cancers as the predominant malignancies, consistent with national and global trends (1, 2), alongside significant burdens of NPC, leukemia, and lymphoma. Crude cancer incidence in Zhanjiang City rose 2.32% annually, exceeding the National Cancer Center’s 1.4% benchmark. A substantial comorbidity burden was evident, with a median of 5 comorbidities per patient, varying markedly by cancer type. Patients with liver, lung, and nasopharyngeal cancers bore the heaviest disease burden. Crucially, association rule mining revealed distinct, malignancy-specific comorbidity clusters, particularly involving liver-hepatic, lung-respiratory, and colorectal-inflammatory pathways. These patterns exhibited significant heterogeneity across sex, age groups, and urban-rural residence, reflecting underlying biological, behavioral, and healthcare access factors.

### Comorbidity burden and profiles

Geriatric populations bear the greatest cancer burden, with over half of cancer diagnoses occurring at age 65 or older according to established literature (24). Our data corroborate this pattern, showing initial diagnoses at ≥60 and ≥65 years in 60.12% and 47.94% of cases, respectively. Age-related cumulative cellular damage and physiological decline predispose elderly cancer patients to comorbid noncommunicable conditions through degenerative pathways (25). Comorbidity prevalence among cancer patients exhibits substantial methodological heterogeneity globally, with reported rates ranging widely from 0.4% to 90% across studies due to variations in measurement approaches, population characteristics, and malignancy types (26). Geriatric-focused studies and clinical chart reviews consistently report higher burdens than analyses of administrative databases; for instance, SEER data indicate 68.7% prevalence while NHIS reports show 43.7% to 46.6%, dominated by hypertension, arthritis, cardiovascular disease, diabetes, and respiratory disorders (27, 28). Crucially, our investigation utilized clinician-curated diagnostic data from medical institutions, offering superior diagnostic accuracy compared to public databases or self-reported sources. This robust methodology revealed elevated comorbidity rates, with a median of 5 comorbidities per patient and a mean prevalence of 88.3% across malignancies. These findings align with established evidence of site-specific variation, where U.S. cohorts demonstrate comorbidity prevalence ranging from 40.2% to 78.7%. Notably, liver cancer demonstrated the highest comorbidity burden at 97.26%, followed by lung, prostate, and esophageal malignancies, while breast, skin, and thyroid cancers exhibited lower burdens. This hierarchy reflects the pathophysiology whereby visceral malignancies such as hepatic and pulmonary cancers drive age-dependent comorbidity escalation through chronic organ dysfunction, manifesting clinically as hypoproteinemia, ascites, atelectasis, and malnutrition (29, 30). In contrast, superficial carcinomas incur substantially reduced comorbidity risks due to their minimal functional impairment.

### Cancer-specific comorbidity heterogeneity

#### Colorectal cancer

A retrospective cohort study of 5,312 patients using latent class analysis showed that 59% had at least one comorbidity, with 19% of the cohort presenting four or more comorbidities. The median comorbidity count reached five conditions, and 80% of patients exhibited three or more concurrent conditions. Cardiovascular/respiratory diseases, diabetes, metabolic disorders, anemia, and gastrointestinal pathologies emerged as the most prevalent comorbidities according to reference (31). Observed discrepancies between studies primarily stem from methodological differences, particularly variations in assessment tools such as the Charlson Index versus more comprehensive diagnostic criteria.

#### Breast cancer

Analysis of South African data from 2,281 participants documented a 44% comorbidity prevalence. The most frequent conditions included obesity at 52.8%, hypertension affecting 41.3% of cases, HIV present in 22.0%, and diabetes observed in 13.7% of the cohort according to reference (32). Our larger study of 6,956 patients demonstrated a significantly higher prevalence of 74.1%, primarily driven by benign breast dysplasia, hepatic cysts, anemia, hypertension, and diabetes. The association with benign breast disease should be interpreted cautiously, as it represents a recognized risk or precursor state rather than a strict comorbidity and may reflect antecedent disease history or surveillance-related coding in inpatient claims. Hormonal pathways such as estrogen-mediated obesity and dysplasia may contribute to this distinct clinical profile, underscoring the need for integrated management approaches.

#### Lung cancer

Significant prevalence heterogeneity reflected methodological diversity (33–36). Common conditions included pneumonia, cerebral infarction, hypertension, diabetes, and COPD (37–40). Our analysis identified 32 distinct comorbidities, with pneumonia, pleural diseases, atelectasis, hypertension, COPD, and diabetes predominating. Association rules revealed 23 significant combinations, notably COPD-pleural disease-hypertension and ischemic cardiomyopathy pneumonia clusters.

### Comorbidity profiles by demographic stratification

Comorbidity patterns exhibited significant age-and sex-stratified heterogeneity. Network analysis of 8.8 million discharge records established demographic-specific variations with differential clinical impacts (41). Our association rule mining confirmed divergent comorbidity patterns across sex/age groups in malignant neoplasms, attributable to multifactorial interactions including physiological dimorphism, endocrine regulation, genetic susceptibility, lifestyle behaviors, and environmental exposures.

The shared burden of anemia and metabolic disorders likely reflects systemic effects of cancer and cytotoxic therapies. The male predominance in hepatopulmonary comorbidities aligns with higher rates of smoking and hepatitis exposure among males. However, lifestyle factors such as smoking and alcohol use are not captured in our claims dataset. Given the strong sex differences in smoking prevalence and its established role in both lung cancer and respiratory infections, the observed male-specific respiratory co-diagnosis patterns may be partly influenced by unmeasured confounding. Conversely, the female predominance in thyroid/breast comorbidities suggests influences of sex hormones and gender-specific healthcare utilization patterns.

Comorbidity profiles demonstrate progressive transitions across age groups. Younger patients exhibit predominantly cancer-specific complications such as leukemia-related cytopenias and opportunistic infections. Middle-aged cohorts manifest transitional patterns featuring early chronic conditions alongside malignancy-related sequelae. Older adults develop multimorbidity reflective of cumulative organ dysfunction and age-associated chronic diseases including cardiovascular, metabolic, and respiratory pathologies.

Urban-rural divergence primarily stems from differential malignancy prevalence–particularly elevated hepatocellular carcinoma and NPC burdens in rural areas potentially associated with viral exposures—coupled with healthcare access inequities cited. Rural patients demonstrate stronger associations with conditions linked to diagnostic delays or advanced disease presentations, including hepatic fibrosis and pneumonia. Urban cohorts more frequently exhibit conditions amenable to chronic management such as ischemic heart disease and benign breast disorders.

### Advantages and limitations

This study’s strengths include comprehensive analysis of six-year, multi-hospital insurance claims data (n=163), enhancing accuracy in characterizing cancer comorbidity patterns. Several limitations should be acknowledged. This study relied on inpatient administrative insurance claims data, which are subject to diagnostic misclassification and inter-hospital coding variability. The lack of longitudinal follow-up and outpatient records limited the assessment of disease trajectories. Moreover, key clinical variables, including cancer stage, disease severity, laboratory results, treatment details, and behavioral factors such as smoking and alcohol consumption, were unavailable, restricting clinical stratification and increasing the risk of residual confounding. As temporality between cancer and co-recorded conditions cannot be established from inpatient claims alone, the observed associations should be interpreted as co-occurrence patterns rather than causal relationships. Finally, the regional nature of the dataset may limit the generalizability of these findings.

## Conclusions

This large-scale analysis provides a detailed characterization of the malignant neoplasm spectrum and reveals complex, clinically significant comorbidity patterns among hospitalized patients. We confirm a substantial comorbidity burden, particularly associated with lung, liver, and colorectal cancers, and demonstrate distinct patterns using association rule mining. The significant variations observed across sex, age, and urban-rural residence underscore the influence of demographic and potentially socio-environmental factors on comorbid disease presentation. These findings highlight the critical need for integrated clinical management strategies that address both cancer and concurrent chronic conditions. Optimizing resource allocation towards populations with the highest comorbidity burden with older adults, males, rural residents and those with specific high-risk clusters is essential. The identified comorbidity patterns offer valuable insights for developing tailored approaches to cancer care that extend beyond the specific geographic origin of the data used in this analysis. Future research should focus on validating these patterns in diverse populations, elucidating underlying mechanisms, and evaluating interventions to improve outcomes for cancer patients with comorbidities.

## Data Availability

The original contributions presented in the study are included in the article/**Supplementary Material**. Further inquiries can be directed to the corresponding authors.
