# Supplementary material for: Disease spectrum and comorbidity patterns of malignant neoplasms: a multi-center hospital-based retrospective analysis of inpatient insurance claims data
Source: Front Oncol. 2026 Mar 27;16:1744397. doi: 10.3389/fonc.2026.1744397 (PMC13065719; doi:10.3389/fonc.2026.1744397)
Supplement: Supplementary file 1 [file Presentation1.pdf]

S1 Table Distribution of included hospitals by level

Level	Number	Percentage(%)
Tertiary hospitals	5	3.0
Secondary hospitals	49	30.1
Primary hospitals	109	66.9
Total	163	100.0

S2 Table ICD-10 codes corresponding to comorbidity categories displayed in Figure 5

Comorbidity Category (Figure 5)	ICD-10 Codes
Acute upper respiratory infection	J00_J06
Agranulocytosis	D70
Anaemia	D50_D64
Arrhythmia	I47_I49
Ascites	R18
Benign mammary dysplasia	N60
Cerebral infarction	I63
Cholecystitis	K81
Chronic ischaemic heart disease	I25
Chronic obstructive pulmonary disease	J44
Chronic sinusitis	J32
Chronic viral hepatitis	B18
Chronic rhinitis, nasopharyngitis and pharyngitis	J31
Dermatophytosis	B35
Diabetes mellitus	E10_E14
Diseases of spleen	D73
Emphysema	J43
Essential (primary) hypertension	I10
Fibrosis and cirrhosis of liver	K74
Gastritis and duodenitis	K29
Gastrointestinal haemorrhage, unspecified	K92
Haemorrhoids	I84
Heart failure	I50
Hepatic failure	K72
Hyperplasia of prostate	N40
Leiomyoma of uterus	D25
Malignant neoplasm of bronchus and lung	C33_C34
Mastoiditis	H70
Metabolic disorders	E70_E90
Nontoxic goitre	E04
Other cerebrovascular diseases	I67
Other diseases of anus and rectum	K62

Other diseases of biliary tract	K83
Other diseases of gallbladder	K82
Other diseases of intestine	K63
Other diseases of liver	K76
Other diseases of pericardium	I31
Other diseases of stomach and duodenum	K31
Other disorders of brain	G93
Other disorders of kidney and ureter	N28
Other disorders of urinary system	N39
Other noninfectious gastroenteritis and colitis	K52
Other respiratory disorders	J98
Paralytic ileus	K56
Pleural condition	J94
Pneumonia	J12_J18
Purpura and other haemorrhagic conditions	D69
Renal failure	N17_N19
Respiratory failure	J96
Septicaemia	A41
Spondylopathies	M45_M49
Systemic Inflammatory Response Syndrome	R65
Toxic liver disease	K71
Tubulo-interstitial nephritis	N10_N12
Urolithiasis	N20_N23

S3 Table The top 20 most frequent comorbidities for each of the 10 most prevalent malignancies

Site	**Comorbidity**	Cases(n)	Prevalence
Lung		23202	
	Pneumonia	10581	45.6%
	Metabolic disorders	7493	32.3%
	Pleural condition	6024	26.0%
	Other respiratory disorders	5888	25.4%
	Essential (primary) hypertension	4928	21.2%
	Ohronic obstructive pulmonary disease	3629	15.6%
	Gastritis and duodenitis	3308	14.3%
	Anaemia	3219	13.9%
	Heart failure	2770	11.9%
	Cerebral infarction	2717	11.7%
	Other diseases of liver	2619	11.3%
	Chronic ischaemic heart disease	2531	10.9%
	Other disorders of kidney and ureter	2276	9.8%
	Arrhythmia	2068	8.9%
	Diabetes mellitus	1976	8.5%

	Emphysema	1797	7.7%
	Hyperplasia of prostate	1642	7.1%
	Respiratory failure	1416	6.1%
	Other cerebrovascular diseases	1361	5.9%
	Other diseases of pericardium	1340	5.8%
Liver		12886	
	Fibrosis and cirrhosis of liver	6022	46.7%
	Chronic viral hepatitis	4707	36.5%
	Metabolic disorders	4374	33.9%
	Other diseases of liver	2583	20.0%
	Ascites	2559	19.9%
	Anaemia	2388	18.5%
	Cholelithiasis	2159	16.8%
	Gastritis and duodenitis	2018	15.7%
	Pneumonia	2015	15.6%
	Other disorders of kidney and ureter	1898	14.7%
	Essential (primary) hypertension	1702	13.2%
	Other respiratory disorders	1438	11.2%
	Hepatic failure	1331	10.3%
	Diabetes mellitus	1182	9.2%
	Pleural condition	1119	8.7%
	Cholecystitis	1057	8.2%
	Gastrointestinal haemorrhage, unspecified	1001	7.8%
	Other diseases of biliary tract	973	7.6%
	Diseases of spleen	793	6.2%
	Urolithiasis	775	6.0%
Colorectum		12657	
	Metabolic disorders	3764	29.7%
	Anaemia	3253	25.7%
	Paralytic ileus	2388	18.9%
	Essential (primary) hypertension	2279	18.0%
	Other diseases of intestine	2176	17.2%
	Other disorders of kidney and ureter	2107	16.6%
	Other diseases of liver	2043	16.1%
	Gastritis and duodenitis	2020	16.0%
	Pneumonia	1969	15.6%
	Haemorrhoids	1375	10.9%
	Diabetes mellitus	1178	9.3%
	Cholelithiasis	1159	9.2%
	Hyperplasia of prostate	1088	8.6%
	Other respiratory disorders	1057	8.4%
	Chronic ischaemic heart disease	917	7.2%
	Arrhythmia	916	7.2%
	Urolithiasis	864	6.8%

	Agranulocytosis	821	6.5%
	Other diseases of anus and rectum	821	6.5%
	Heart failure	756	6.0%
Breast		6956	
	Metabolic disorders	1703	24.5%
	Benign mammary dysplasia	1455	20.9%
	Other diseases of liver	1197	17.2%
	Anaemia	1085	15.6%
	Agranulocytosis	883	12.7%
	Essential (primary) hypertension	833	12.0%
	Pneumonia	771	11.1%
	Nontoxic goitre	691	9.9%
	Diabetes mellitus	510	7.3%
	Leiomyoma of uterus	492	7.1%
	Other respiratory disorders	489	7.0%
	Toxic liver disease	389	5.6%
	Pleural condition	364	5.2%
	Arrhythmia	343	4.9%
	Other disorders of kidney and ureter	342	4.9%
	Cholelithiasis	309	4.4%
	Gastritis and duodenitis	252	3.6%
	Urolithiasis	235	3.4%
	Other diseases of gallbladder	235	3.4%
	Hepatic failure	207	3.0%
Thyroid		5897	
	Nontoxic goitre	1621	27.5%
	Metabolic disorders	737	12.5%
	Essential (primary) hypertension	662	11.2%
	Other diseases of liver	622	10.5%
	Pneumonia	335	5.7%
	Diabetes mellitus	310	5.3%
	Other respiratory disorders	277	4.7%
	Other disorders of kidney and ureter	227	3.8%
	Gastritis and duodenitis	227	3.8%
	Arrhythmia	207	3.5%
	Other diseases of gallbladder	206	3.5%
	Anaemia	198	3.4%
	Leiomyoma of uterus	187	3.2%
	Cholelithiasis	170	2.9%
	Urolithiasis	170	2.9%
	Cerebral infarction	141	2.4%
	Chronic ischaemic heart disease	132	2.2%
	Chronic rhinitis, nasopharyngitis and pharyngitis	130	2.2%
	Malignant neoplasm of bronchus and lung	126	2.1%

	Heart failure	114	1.9%
NPC		5785	
	Metabolic disorders	2142	37.0%
	Chronic sinusitis	1883	32.5%
	Agranulocytosis	1429	24.7%
	Anaemia	1383	23.9%
	Pneumonia	1055	18.2%
	Acute upper respiratory infection	991	17.1%
	Mastoiditis	811	14.0%
	Other diseases of liver	773	13.4%
	Other respiratory disorders	680	11.8%
	Essential (primary) hypertension	613	10.6%
	Other disorders of brain	524	9.1%
	Toxic liver disease	478	8.3%
	Other disorders of kidney and ureter	444	7.7%
	Chronic viral hepatitis	433	7.5%
	Gastritis and duodenitis	428	7.4%
	Arrhythmia	386	6.7%
	Chronic rhinitis, nasopharyngitis and pharyngitis	383	6.6%
	Purpura and other haemorrhagic conditions	352	6.1%
	Cholelithiasis	297	5.1%
	Nontoxic goitre	296	5.1%
Stomach		5673	
	Anaemia	2292	40.4%
	Metabolic disorders	1867	32.9%
	Gastritis and duodenitis	1330	23.4%
	Other disorders of kidney and ureter	886	15.6%
	Pneumonia	884	15.6%
	Other diseases of stomach and duodenum	864	15.2%
	Gastrointestinal haemorrhage, unspecified	838	14.8%
	Other diseases of liver	718	12.7%
	Essential (primary) hypertension	683	12.0%
	Ascites	569	10.0%
	Other respiratory disorders	568	10.0%
	Chronic ischaemic heart disease	490	8.6%
	Cholelithiasis	447	7.9%
	Heart failure	438	7.7%
	Arrhythmia	383	6.8%
	Pleural condition	381	6.7%
	Hyperplasia of prostate	364	6.4%
	Paralytic ileus	353	6.2%
	Diabetes mellitus	329	5.8%
	Urolithiasis	310	5.5%
Cervix		3671	

	Anaemia	1395	37.9%
	Metabolic disorders	1029	28.0%
	Agranulocytosis	692	18.8%
	Essential (primary) hypertension	571	15.5%
	Other diseases of liver	533	14.5%
	Other disorders of urinary system	532	14.5%
	Leiomyoma of uterus	398	10.8%
	Tubulo-interstitial nephritis	376	10.2%
	Other respiratory disorders	349	9.5%
	Pneumonia	343	9.3%
	Other disorders of kidney and ureter	342	9.3%
	Diabetes mellitus	314	8.5%
	Renal failure	235	6.4%
	Other noninfective gastroenteritis and colitis	207	5.6%
	Cholelithiasis	194	5.3%
	Arrhythmia	191	5.2%
	Gastritis and duodenitis	184	5.0%
	Urolithiasis	171	4.7%
	Other diseases of anus and rectum	149	4.1%
	Spondylopathies	133	3.6%
Lymphoma		3616	
	Metabolic disorders	1027	34.4%
	Anaemia	897	28.4%
	Pneumonia	651	24.8%
	Other respiratory disorders	441	18.0%
	Essential (primary) hypertension	430	12.2%
	Agranulocytosis	427	11.9%
	Gastritis and duodenitis	383	11.8%
	Pleural condition	376	10.6%
	Other diseases of liver	333	10.4%
	Other disorders of kidney and ureter	325	9.2%
	Chronic viral hepatitis	289	9.0%
	Heart failure	271	8.0%
	Arrhythmia	268	7.5%
	Diabetes mellitus	224	7.4%
	Purpura and other haemorrhagic conditions	210	6.2%
	Acute upper respiratory infection	206	5.8%
	Cerebral infarction	202	5.7%
	Cholelithiasis	202	5.6%
	Chronic sinusitis	199	5.6%
	Toxic liver disease	199	5.5%
Leukemia		3596	
	Pneumonia	1187	33.00%
	Other respiratory disorders	1043	29.0%

Anaemia	895	24.9%
Metabolic disorders	856	23.8%
Septicaemia	619	17.2%
Heart failure	521	14.5%
Essential (primary) hypertension	403	11.2%
Gastritis and duodenitis	363	10.1%
Acute upper respiratory infection	360	10.0%
Hepatic failure	309	8.6%
Dermatophytosis	295	8.2%
Cerebral infarction	248	6.9%
Other diseases of liver	230	6.4%
Chronic ischaemic heart disease	230	6.4%
Pleural condition	227	6.3%
Diabetes mellitus	219	6.1%
Arrhythmia	191	5.3%
Gastrointestinal haemorrhage, unspecified	187	5.2%
Systemic Inflammatory Response Syndrome	180	5.0%
Purpura and other haemorrhagic conditions	176	4.9%

S4-13 Fig Cancer-specific comorbidity networks: (4) Lung, (5) Liver, (6) Colorectal, (7) Breast, (8) Thyroid, (9) NPC, (10) Gastric, (11) Cervical, (12) Lymphoma, (13) Leukemia.

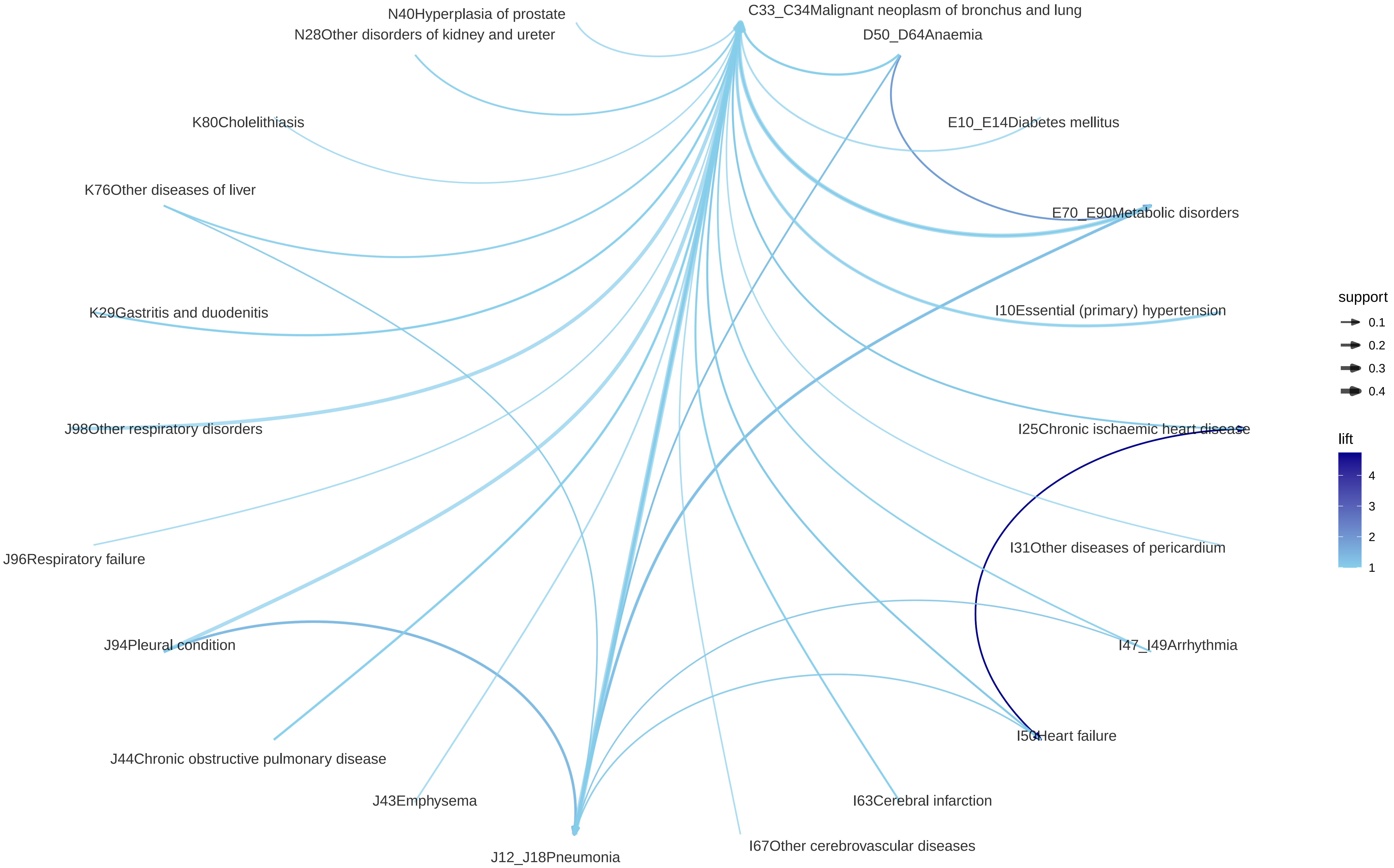
N40Hyperplasia of prostate
C33_C34Malignant neoplasm of bronchus and lung
N28Other disorders of kidney and ureter
D50_D64Anaemia
K80Cholelithiasis
E10_E14Diabetes mellitus
K76Other diseases of liver
E70_E90Metabolic disorders
K29Gastritis and duodenitis
I10Essential (primary) hypertension
J98Other respiratory disorders
I25Chronic ischaemic heart disease
J96Respiratory failure
I31Other diseases of pericardium
J94Pleural condition
I47_I49Arrhythmia
J44Chronic obstructive pulmonary disease
I50Heart failure
J43Emphysema
I63Cerebral infarction
J12_J18Pneumonia
I67Other cerebrovascular diseases
support
0.1
0.2
0.3
0.4
lift
4
3
2
1

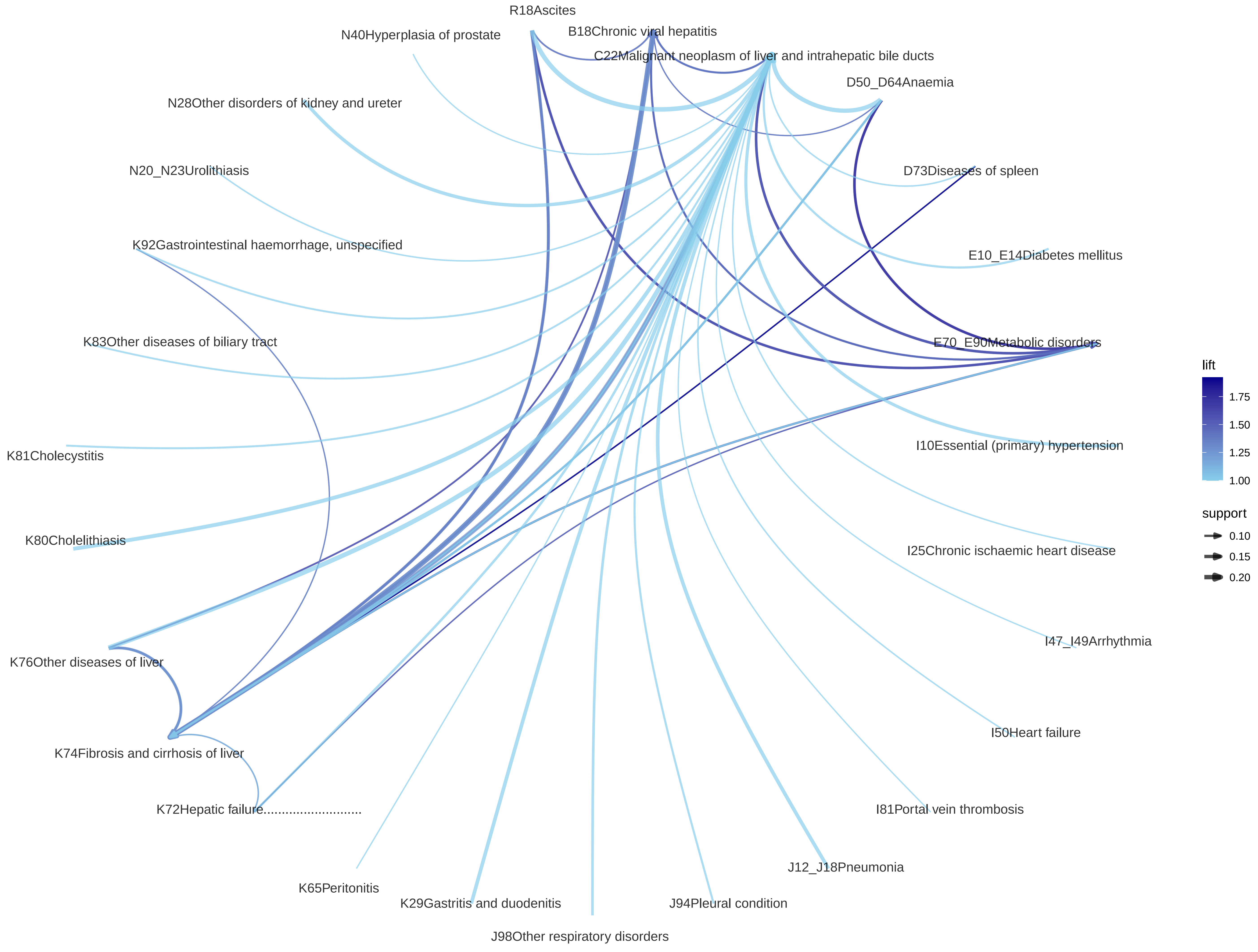
R18Ascites
N40Hyperplasia of prostate
B18Chronic viral hepatitis
C22Malignant neoplasm of liver and intrahepatic bile ducts
D50_D64Anaemia
N28Other disorders of kidney and ureter
N20_N23Urolithiasis
D73Diseases of spleen
K92Gastrointestinal haemorrhage, unspecified
E10_E14Diabetes mellitus
K83Other diseases of biliary tract
E70_E90Metabolic disorders
K81Cholecystitis
I10Essential (primary) hypertension
K80Cholelithiasis
I25Chronic ischaemic heart disease
K76Other diseases of liver
I47_I49Arrhythmia
K74Fibrosis and cirrhosis of liver
I50Heart failure
K72Hepatic failure..........................
I81Portal vein thrombosis
K65Peritonitis
J12_J18Pneumonia
K29Gastritis and duodenitis
J94Pleural condition
J98Other respiratory disorders
lift
1.75
1.50
1.25
1.00
support
0.10
0.15
0.20

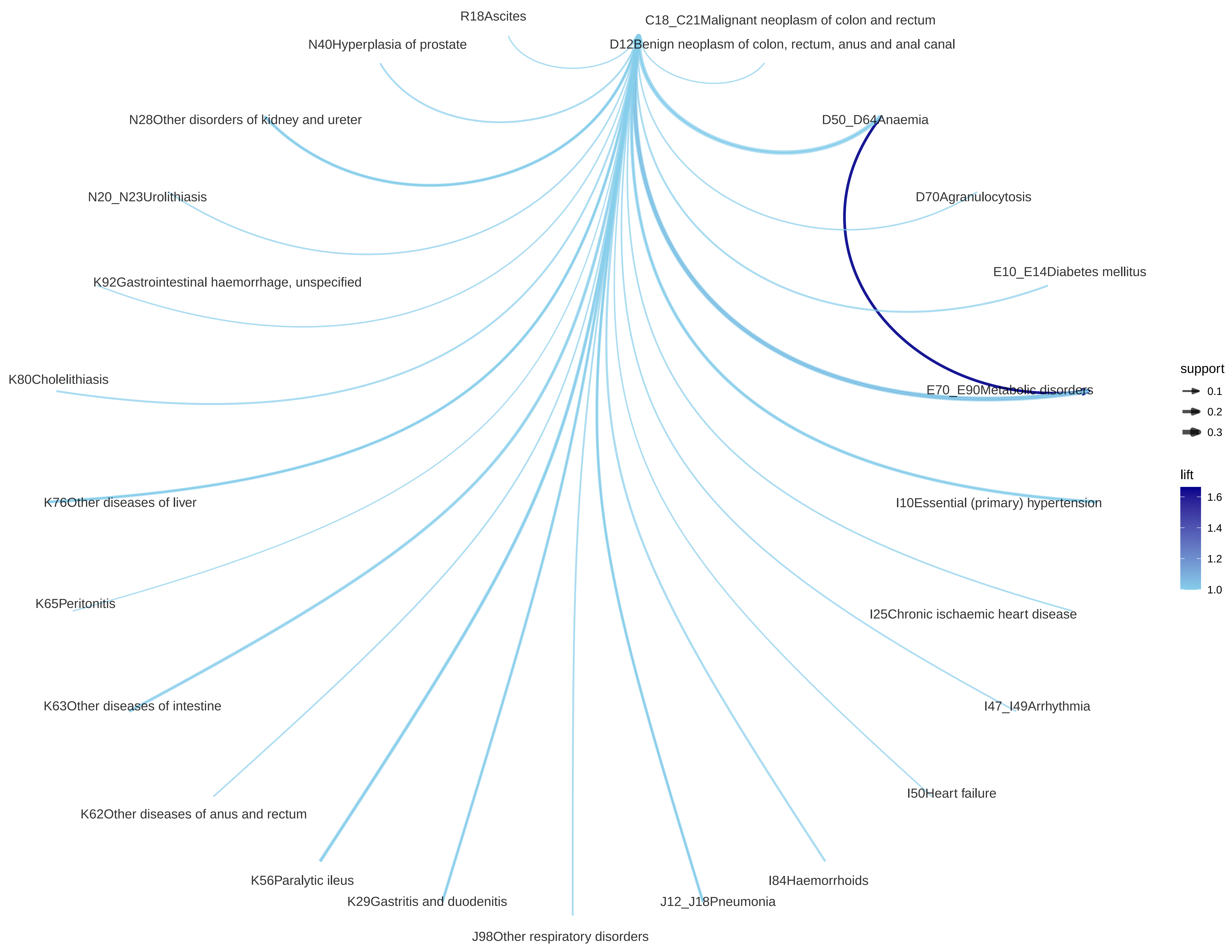
R18Ascites
C18_C21Malignant neoplasm of colon and rectum
N40Hyperplasia of prostate
D12Benign neoplasm of colon, rectum, anus and anal canal
N28Other disorders of kidney and ureter
D50_D64Anaemia
N20_N23Urolithiasis
D70Agranulocytosis
K92Gastrointestinal haemorrhage, unspecified
E10_E14Diabetes mellitus
K80Cholelithiasis
E70_E90Metabolic disorders
support
0.1
0.2
0.3
lift
1.6
1.4
1.2
1.0
K76Other diseases of liver
I10Essential (primary) hypertension
K65Peritonitis
I25Chronic ischaemic heart disease
K63Other diseases of intestine
I47_I49Arrhythmia
I50Heart failure
K62Other diseases of anus and rectum
K56Paralytic ileus
I84Haemorrhoids
K29Gastritis and duodenitis
J12_J18Pneumonia
J98Other respiratory disorders

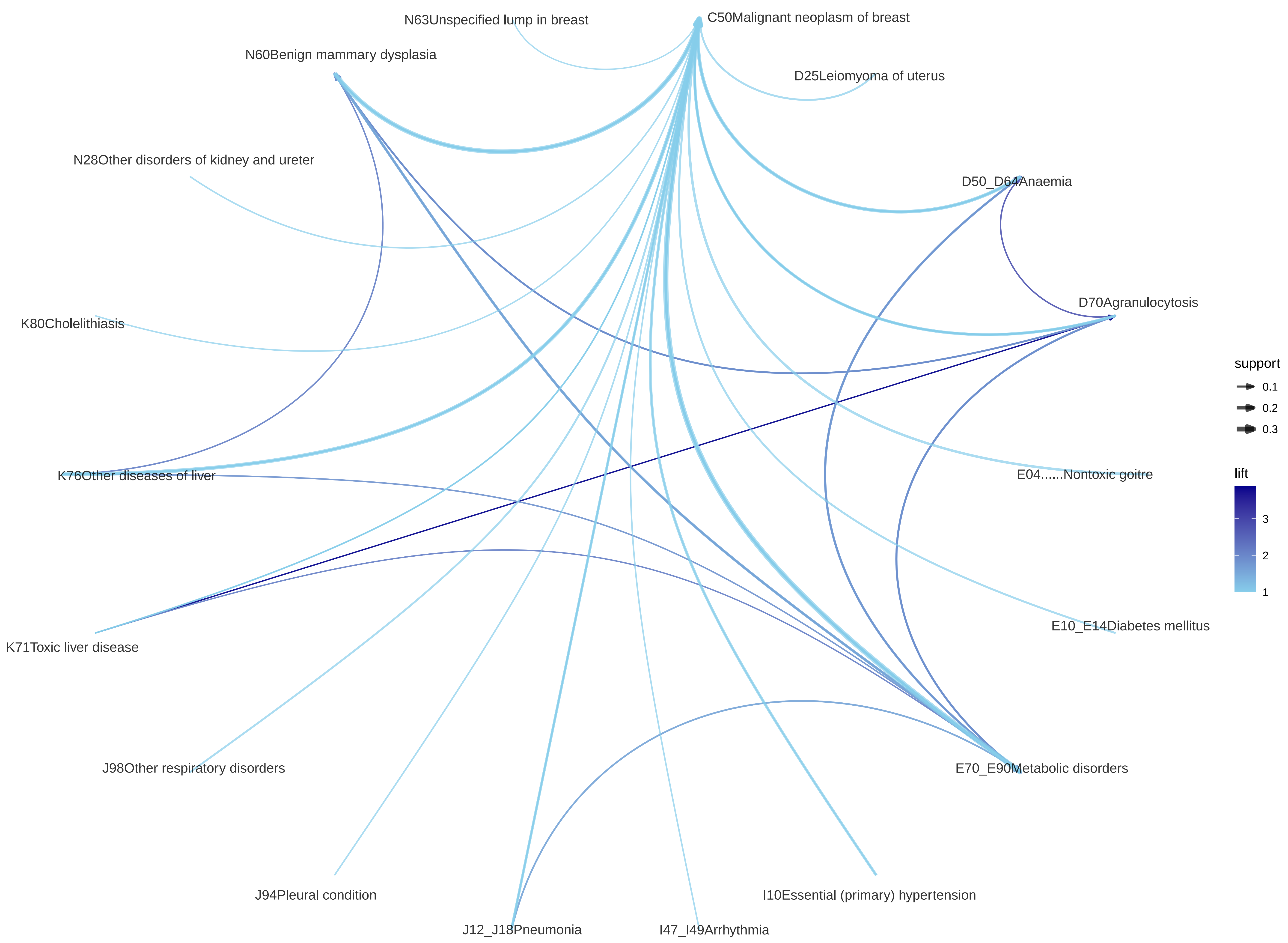

N63Unspecified lump in breast
C50Malignant neoplasm of breast
N60Benign mammary dysplasia
D25Leiomyoma of uterus
N28Other disorders of kidney and ureter
D50_D64Anaemia
D70Agranulocytosis
K80Cholelithiasis
support
0.1
0.2
0.3
K76Other diseases of liver
E04......Nontoxic goitre
lift
3
2
1
E10_E14Diabetes mellitus
K71Toxic liver disease
J98Other respiratory disorders
E70_E90Metabolic disorders
J94Pleural condition
I10Essential (primary) hypertension
J12_J18Pneumonia
I47_I49Arrhythmia

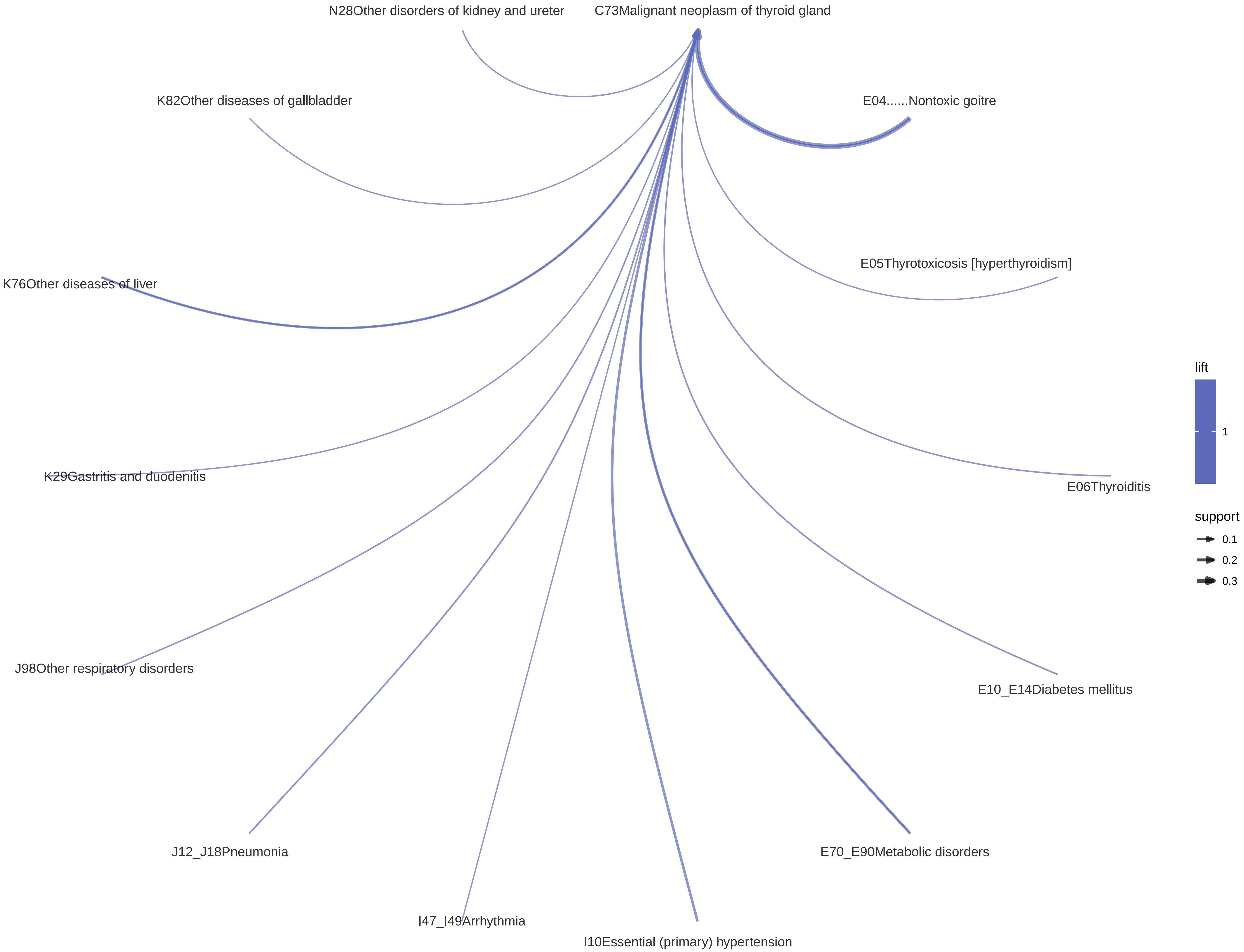

N28Other disorders of kidney and ureter
C73Malignant neoplasm of thyroid gland
K82Other diseases of gallbladder
E04......Nontoxic goitre
E05Thyrotoxicosis [hyperthyroidism]
K76Other diseases of liver
lift
1
K29Gastritis and duodenitis
E06Thyroiditis
support
0.1
0.2
0.3
J98Other respiratory disorders
E10_E14Diabetes mellitus
J12_J18Pneumonia
E70_E90Metabolic disorders
I47_I49Arrhythmia
I10Essential (primary) hypertension

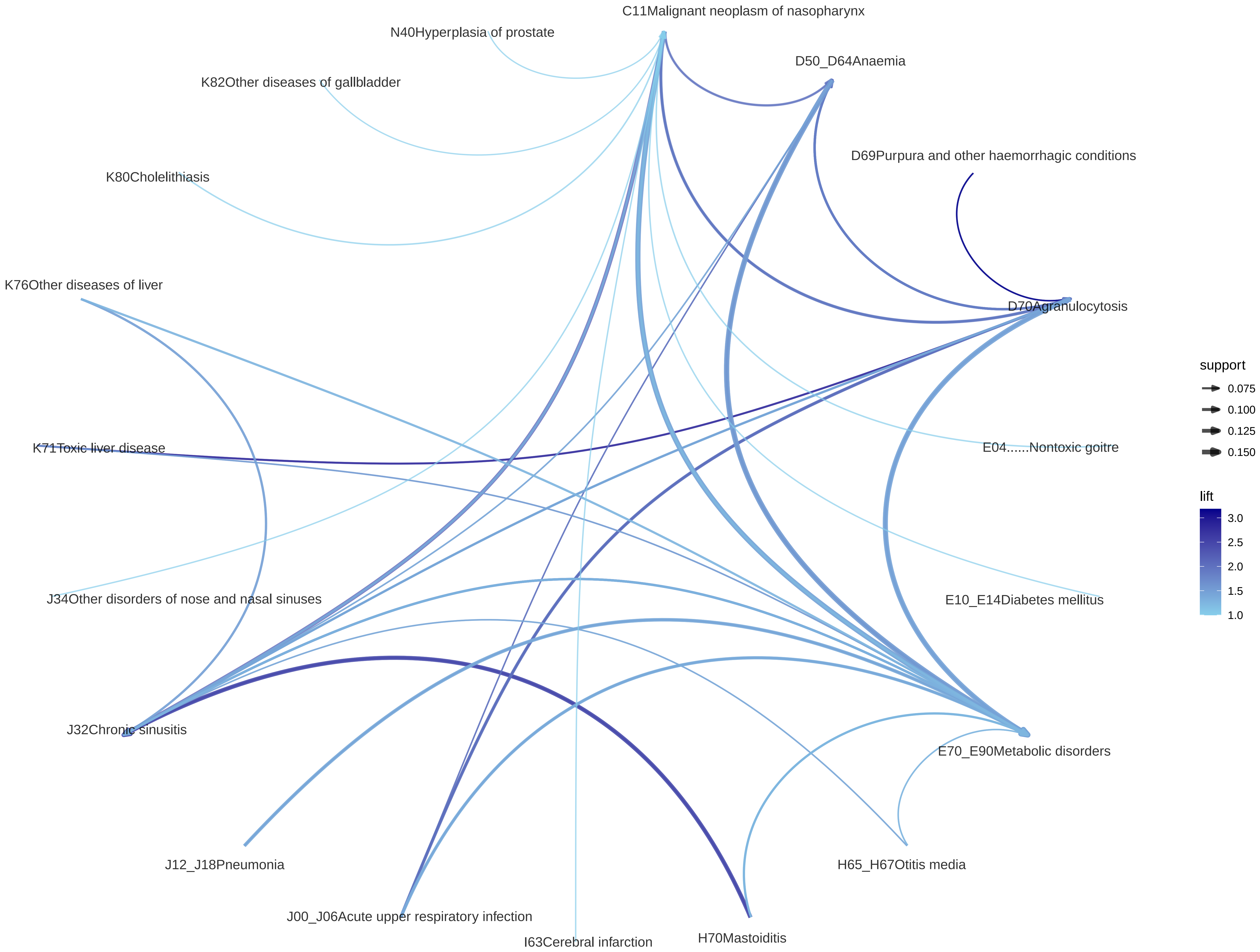
C11Malignant neoplasm of nasopharynx
N40Hyperplasia of prostate
D50_D64Anaemia
K82Other diseases of gallbladder
D69Purpura and other haemorrhagic conditions
K80Cholelithiasis
K76Other diseases of liver
D70Agranulocytosis
support
0.075
0.100
0.125
0.150
K71Toxic liver disease
E04......Nontoxic goitre
lift
3.0
2.5
2.0
1.5
1.0
J34Other disorders of nose and nasal sinuses
E10_E14Diabetes mellitus
J32Chronic sinusitis
E70_E90Metabolic disorders
J12_J18Pneumonia
H65_H67Otitis media
J00_J06Acute upper respiratory infection
I63Cerebral infarction
H70Mastoiditis

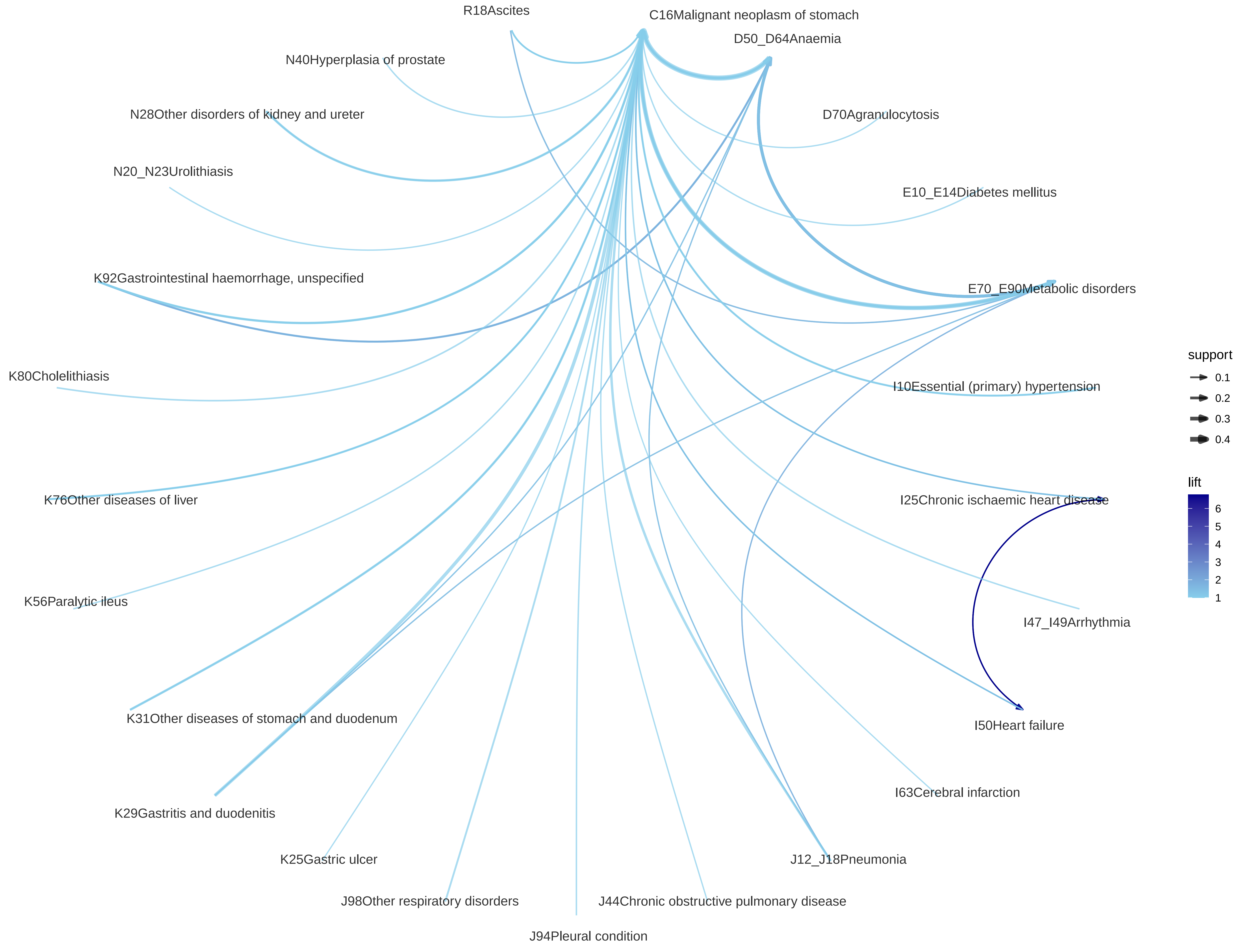
R18Ascites
C16Malignant neoplasm of stomach
D50_D64Anaemia
N40Hyperplasia of prostate
N28Other disorders of kidney and ureter
D70Agranulocytosis
N20_N23Urolithiasis
E10_E14Diabetes mellitus
K92Gastrointestinal haemorrhage, unspecified
E70_E90Metabolic disorders
K80Cholelithiasis
I10Essential (primary) hypertension
K76Other diseases of liver
I25Chronic ischaemic heart disease
K56Paralytic ileus
I47_I49Arrhythmia
K31Other diseases of stomach and duodenum
I50Heart failure
K29Gastritis and duodenitis
I63Cerebral infarction
K25Gastric ulcer
J12_J18Pneumonia
J98Other respiratory disorders
J44Chronic obstructive pulmonary disease
J94Pleural condition
support
0.1
0.2
0.3
0.4
lift
6
5
4
3
2
1

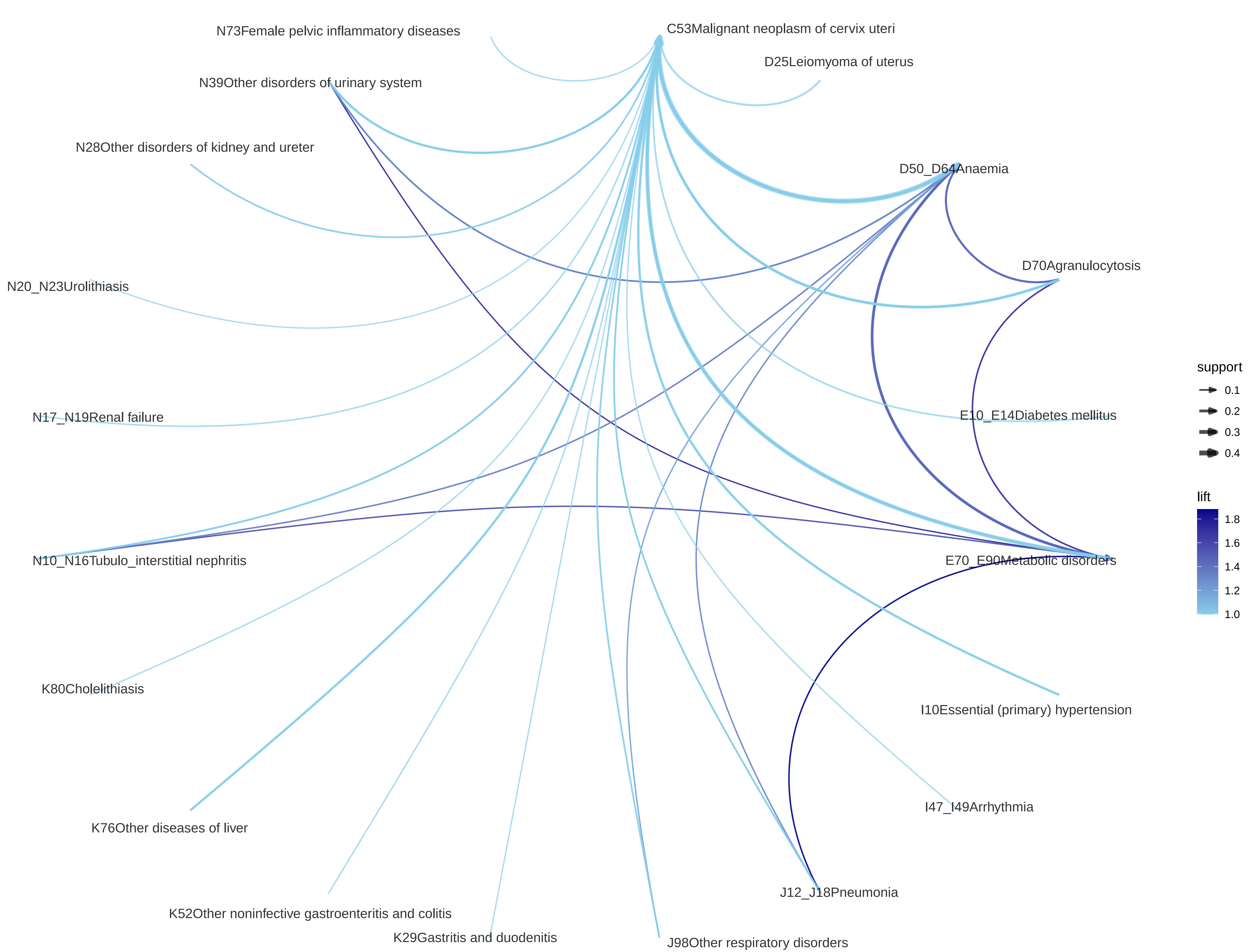

N73Female pelvic inflammatory diseases
C53Malignant neoplasm of cervix uteri
D25Leiomyoma of uterus
N39Other disorders of urinary system
N28Other disorders of kidney and ureter
D50_D64Anaemia
D70Agranulocytosis
N20_N23Urolithiasis
N17_N19Renal failure
E10_E14Diabetes mellitus
N10_N16Tubulo_interstitial nephritis
E70_E90Metabolic disorders
K80Cholelithiasis
I10Essential (primary) hypertension
K76Other diseases of liver
I47_I49Arrhythmia
J12_J18Pneumonia
K52Other noninfective gastroenteritis and colitis
K29Gastritis and duodenitis
J98Other respiratory disorders
support
0.1
0.2
0.3
0.4
lift
1.8
1.6
1.4
1.2
1.0

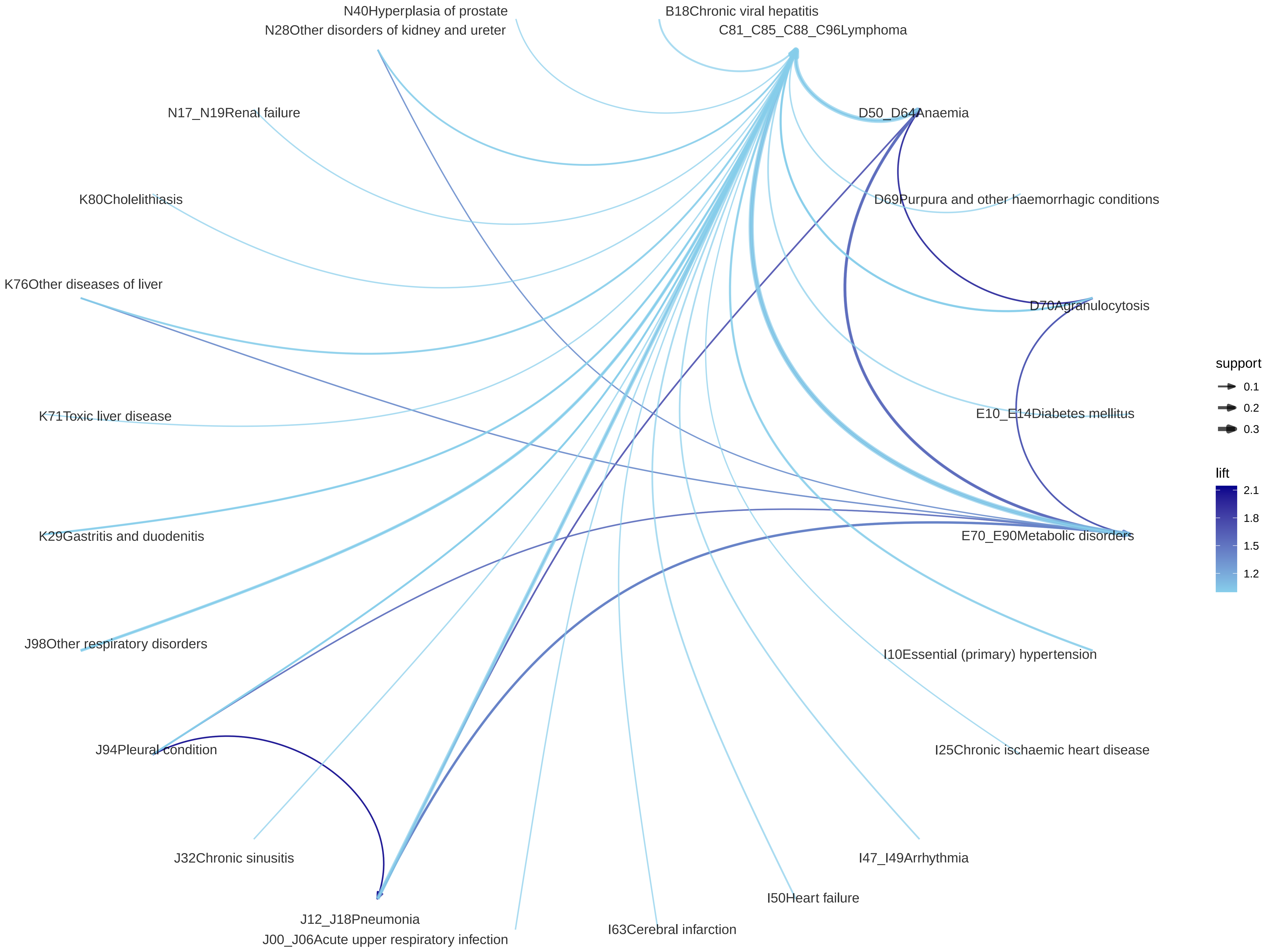
N40Hyperplasia of prostate
B18Chronic viral hepatitis
N28Other disorders of kidney and ureter
C81_C85_C88_C96Lymphoma
N17_N19Renal failure
D50_D64Anaemia
K80Cholelithiasis
D69Purpura and other haemorrhagic conditions
K76Other diseases of liver
D70Agranulocytosis
support
0.1
0.2
0.3
K71Toxic liver disease
E10_E14Diabetes mellitus
lift
2.1
1.8
1.5
1.2
K29Gastritis and duodenitis
E70_E90Metabolic disorders
J98Other respiratory disorders
I10Essential (primary) hypertension
J94Pleural condition
I25Chronic ischaemic heart disease
J32Chronic sinusitis
I47_I49Arrhythmia
I50Heart failure
J12_J18Pneumonia
J00_J06Acute upper respiratory infection
I63Cerebral infarction

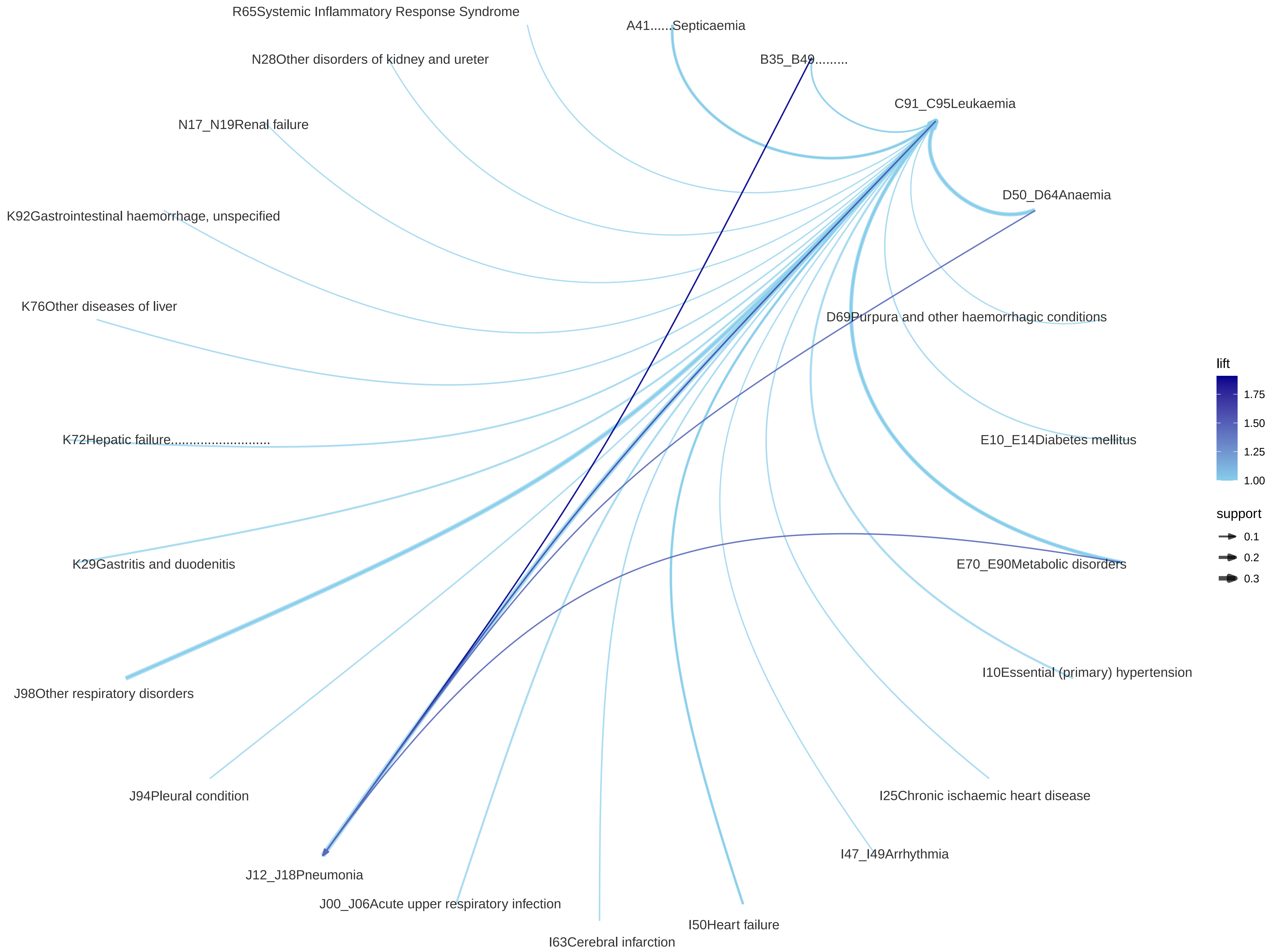
R65Systemic Inflammatory Response Syndrome
A41......Septicaemia
N28Other disorders of kidney and ureter
B35_B49.........
C91_C95Leukaemia
N17_N19Renal failure
D50_D64Anaemia
K92Gastrointestinal haemorrhage, unspecified
K76Other diseases of liver
D69Purpura and other haemorrhagic conditions
lift
1.75
1.50
1.25
1.00
K72Hepatic failure..........................
E10_E14Diabetes mellitus
support
0.1
0.2
0.3
K29Gastritis and duodenitis
E70_E90Metabolic disorders
J98Other respiratory disorders
I10Essential (primary) hypertension
J94Pleural condition
I25Chronic ischaemic heart disease
I47_I49Arrhythmia
J12_J18Pneumonia
J00_J06Acute upper respiratory infection
I50Heart failure
I63Cerebral infarction
